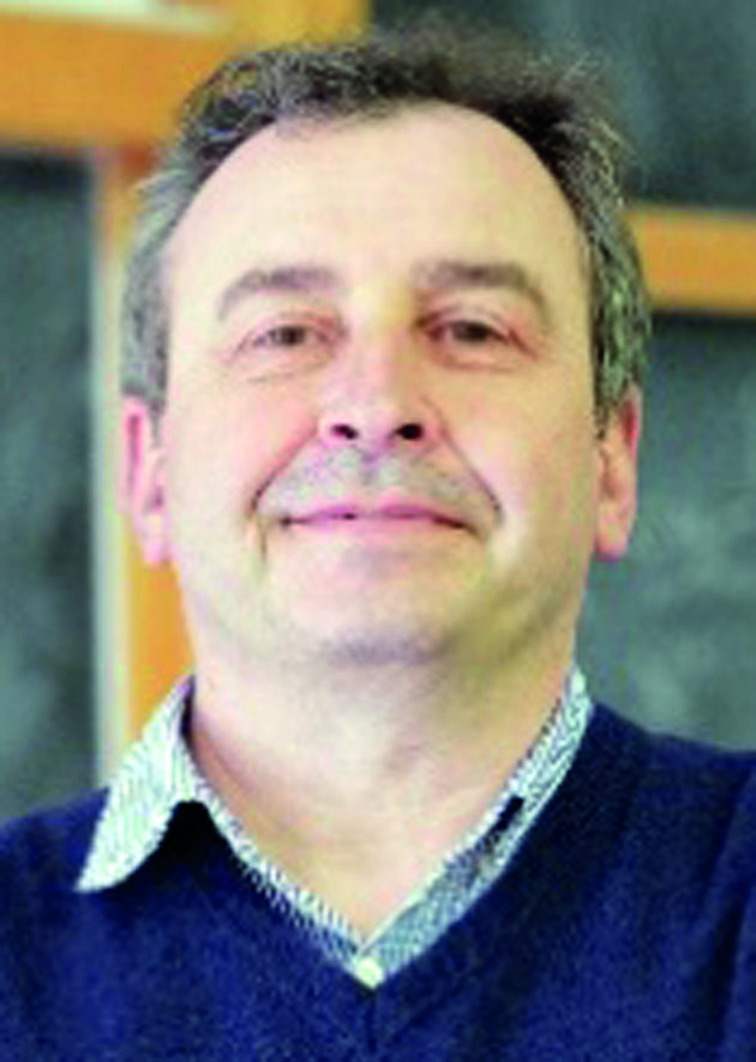# Thermoresponsive Polymers as Viscosity Modifiers: Innovative Nanoarchitectures as Lubricant Additives

**DOI:** 10.1002/cplu.202400611

**Published:** 2024-11-27

**Authors:** Raffaele Carfora, Marcello Notari, Giulio Assanelli, Sara Caramia, Andrea Nitti, Dario Pasini

**Affiliations:** ^1^ Chemistry Department and INSTM Research Unit University of Pavia Via Taramelli 12 27100 Pavia Italy; ^2^ Downstream R&D–Eni S.p.A. Via Felice Maritano 26 20097 San Donato Milanese Italy

**Keywords:** Lubricants, Viscosity modifiers, Stimuli-responsive polymers, Controlled polymerization, Polymer architecture

## Abstract

The world of lubricants is driven by the constant pursuit of improved performance in response of the requests of new engine generations. Engine oils play a critical role as lubricants in mitigating wear, reducing friction and ensuring optimal engine operation under diverse conditions. Modern commercial engine oils are complex formulations, comprising of a base oil, generally coming from petroleum sources, formulated with specific, important additives able to optimize the viscosity, thickening and shear stress in the operating temperature range. Such additives are produced in the thousand tons per year scale range. The most important class of additives for modern lubrication is made of organic polymers with variable architectures and topologies, generally referred as “viscosity modifiers” (VMs): they act as “moderators” of viscosity at different working temperatures. The tremendous advances in polymer science have been reflected in the realm of VMs, allowing the commercialization of products obtained by controlled polymerization techniques, and the experimentation of a broad variety of different macromolecular architectures and topologies as VMs. In this review we introduce the reader, together with the basic principles of viscosity modification and thermal‐dependent rheological response, to the fascinating chemistry towards the improvement of VMs, through optimization of macromolecular design and architecture.

## Graphical abstract

We introduce the reader to the tremendous advances in polymer science reflected in the realm of lubrication, and the experimentation of a broad variety of different macromolecular architectures and topologies as viscosity modifiers.

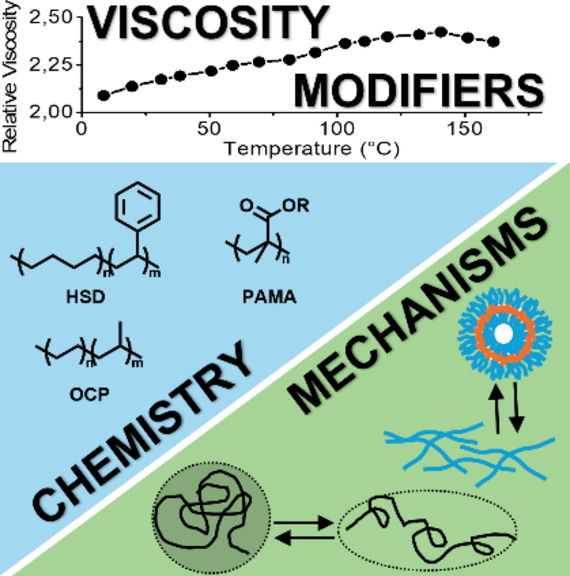

## 1 Introduction

Lubricants are a class of materials used to ensure adequate performance between moving surfaces in terms of reducing friction and wear. Lubricants have been used for hundreds of centuries and are crucial in our life.[^1^, ^2^] There are many examples that illustrate the importance of lubricants in our daily lives: natural lubricants, such as saliva and synovial fluid, lubricate food for easy chewing and reduce joint wear and tear; cooking oils prevent foods from sticking to frying pans and baking trays while also conducting heat.[^3^, ^4^] Historically, ancient Egyptians utilized lubricants to slide large stone block for constructing the great pyramids, while the Romans employed lubricants on the axles of their chariots.[^5^, ^6^] Today, the great demand of non‐aqueous lubricants to ensure the proper functioning of all the machines that permeate our daily lives is met by synthetic formulations derived from petroleum sources.[^7^, ^8^, ^9^]

Modern lubricants are complex formulations able to respond to the diverse issues needed to be addressed in the specific applications, such as transmission fluids,[^10^, ^11^, ^12^] industrial lubricants and automotive or marine engine oils.[^13^, ^14^] They can carry out further functions in addition to lubrication such as cleaning, cooling and sealing.[^15^, ^16^, ^17^]

**Figure 1:**
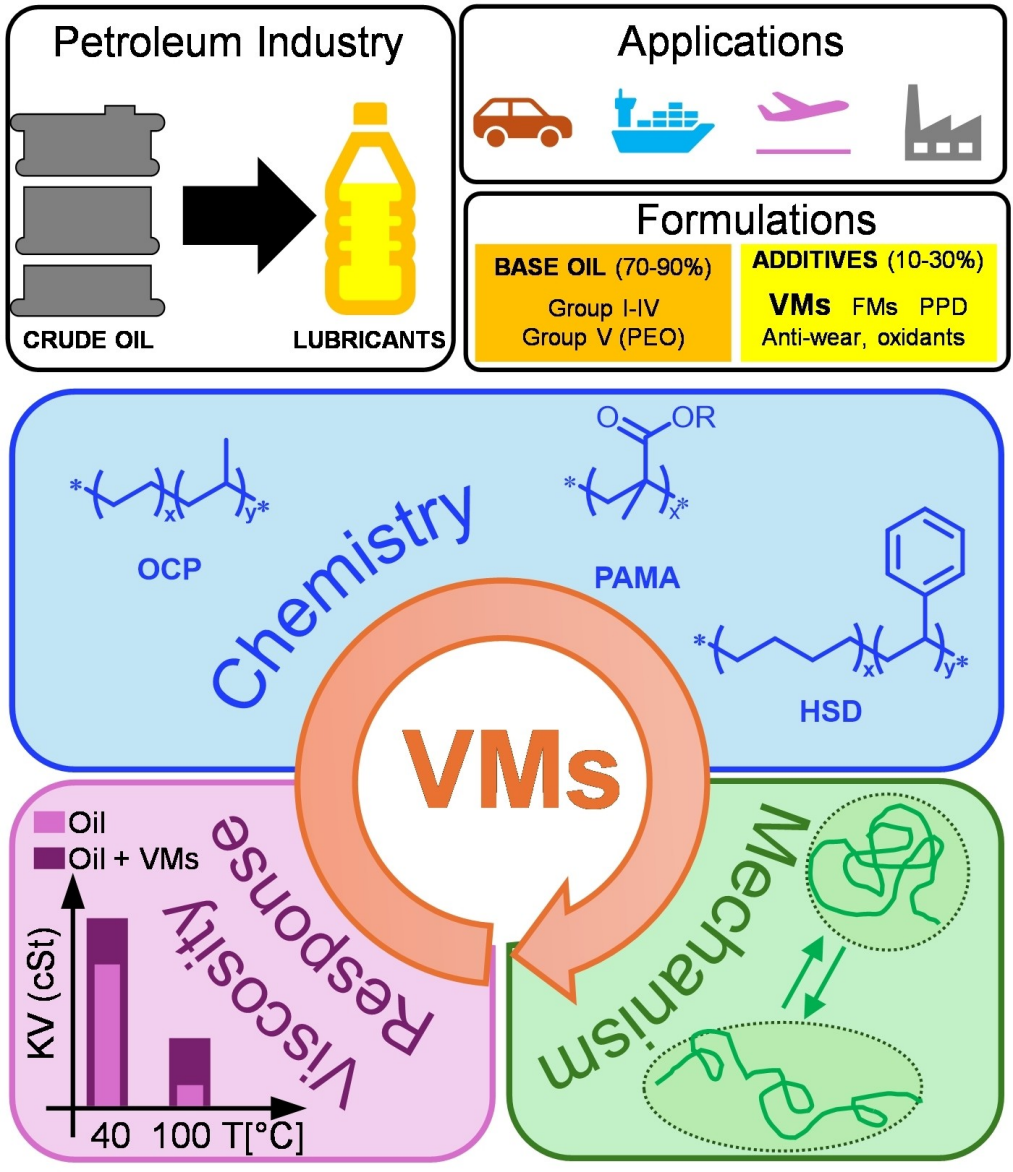
Top: origin, applications and formulations of modern lubricants; bottom: schematic representation of the chemistry and functions of viscosity modifiers (VMs).

Lubricants for the automotive engines are formulation containing 70–90 % of base oils with the remainder being additives (Figure 1).^[18]^ A base oil, typically produced from petroleum crude by vacuum distillation followed by hydro‐processing and hydro‐dewaxing processes, provides the primary vital properties of a lubricant: intrinsic viscosity, thermal stability, low volatility and stability to oxidation.[^19^, ^20^]

Base oils have been classified according to their characteristics (sulfur content and saturates content) and viscosity index (VI) by the API (American Petroleum Institute) into five groups. The first three groups (Group I, II and III) are derived from crude oil, Group IV are based on poly‐α‐olefins (PAOs) and synthesized from the volatile fraction of petroleum. In Group V are included all other mineral and synthetic oils.^[21]^ The base oil serves as the primary fluid medium, providing the necessary viscosity and thickness to reduce friction and wear between moving engine parts, and acts as a heat transfer agent, dissipating heat generated during engine operation. Additionally, the base oils provide a stable platform for additives, that selectively enhance the specific functions of the lubricant, to be effectively dispersed for their optimal functioning (Figure 1). According to their specific functions and performance improvement, the additives are classified in: (1) surface and lubricant protective additives (anti‐wear additives, corrosion inhibitors, antioxidants, dispersants, detergents and antifoams) and (2) performance additives (viscosity modifiers, friction modifiers and pour point depressants).

Surface and lubricant protective additives such as antiwear, corrosion inhibitors, antioxidants, dispersants and detergents extend the life of mechanical components by reducing metal‐to‐metal contact, thereby preventing the corrosion of metal surfaces as well as preventing oxidative reaction that can be altered the chemical composition of formulate.

Performance additives such as friction modifiers (FMs), pour point depressants (PPDs) and viscosity modifiers (VMs) are crucial additives for the optimization of the performance in lubricant formulations. FMs contribute to reducing wear and energy loss by minimizing friction between contacting surfaces. PPDs prevent the solidification of lubricants at low temperatures, maintaining fluidity and preventing mechanical failure.

VMs are high molecular weight polymer‐based additives able to enhance the lubricant's ability to maintain optimal viscosity, shear resistance and shear thickening behavior across a wide temperature range. VMs can be defined as “moderators” of viscosity at different working temperatures. They are essential for a correct operativity of an engine lubricant and representing perhaps the most important class of additives for modern lubrication. The global consumption of lubricant additives in 2020 has been more than 4,000 kton, with VMs leading the market with about 1,000 kton.

The degree of innovation brought about in terms of polymer chemistry in the last few decades is very rich. Different topologies and architectures have been proposed recently in the literature, each of them affecting solution viscosity through different mechanisms. Understanding the correlation between chemical structure, polymer topology and the mechanisms operating in viscosity control is critical to the development of new and more efficient VMs over a wide range of temperatures, also in consideration of the crucial need of an evolution towards increased sustainability.

This work provides an overview of VMs chemistry focusing the attention on the *state‐of‐the‐art* of the materials proposed, as well as novel trends in the field. We have tried to highlight the fundamental connections between the chemistry and the topological aspects of polymers with the mechanisms influencing the viscosity as the macroscopic property.

## 2 Discussion

The metrics used to quantify these properties are briefly presented in section 2.1, together with the main classes of commercial VMs. In section 2.2 we will introduce the mechanisms for viscosity modulation. Finally, in the last section (section 2.3) we will outline the recent developments and the novel trends in the field. In all cases the effect of polymer chemistry on performance metrics is discussed.

### 2.1 Classification, Metrics and Main Classes of VMs

**Figure 2:**
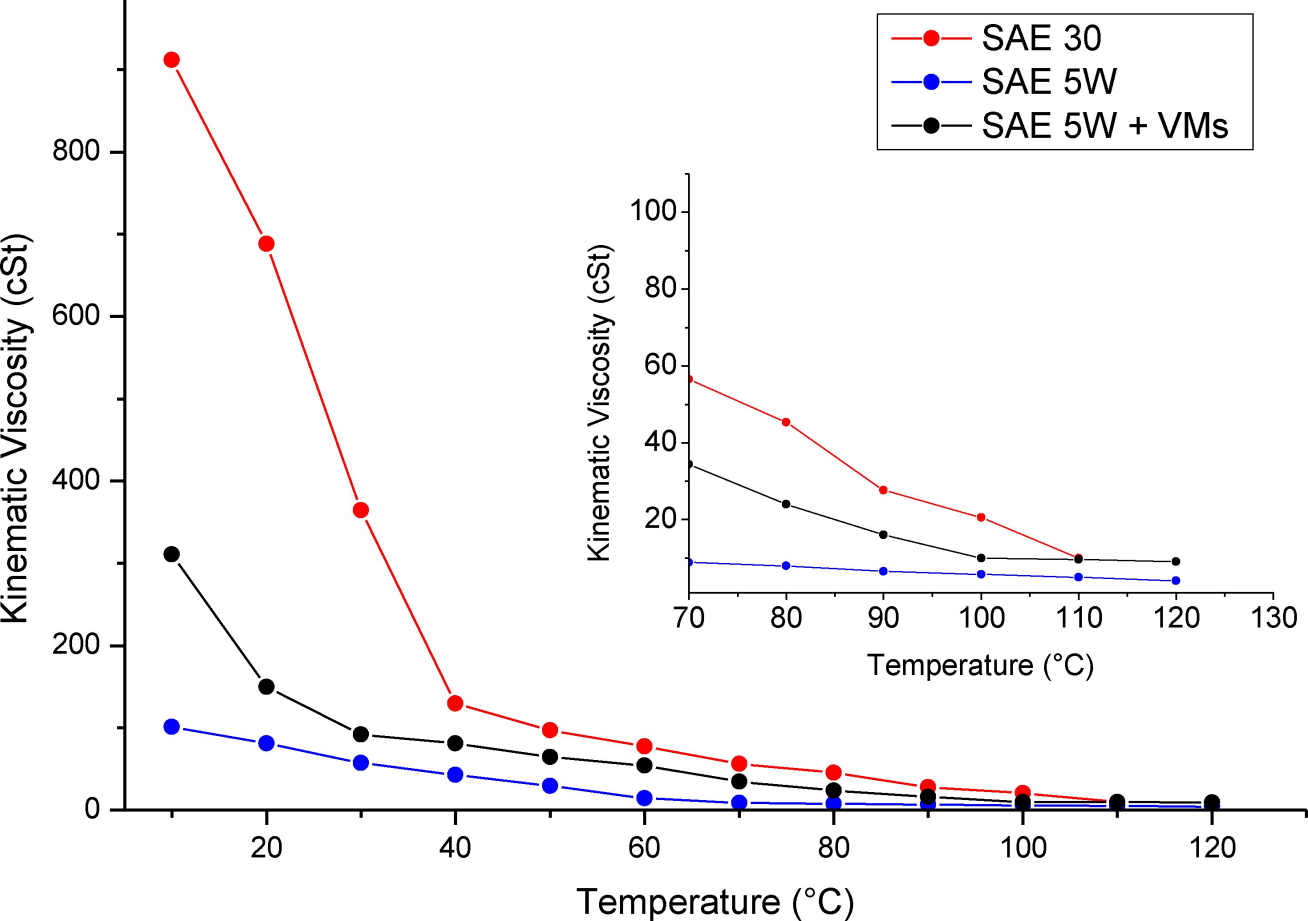
Comparison of two single grade oils, SAE 5 W and SAE 30, and SAE 5 W with PE‐*r*‐PP copolymer as VM. Adapted with permission from ref.^[26]^ Copyright 1991 American Chemical Society.

The three properties by which VMs performances are evaluated are: viscosity‐temperature relationship, thickening efficiency and shear stability. The base oil is defined as “single grade” in the absence of specific additives such as VMs, and in this case the modulation of the viscosity with temperature depends on its chemical structure and molecular weight characteristics. Monograde oils usually exhibit very significant viscosity changes in the temperature range from −40 °C to 150 °C, where typical motor engines operate. Monograde oils are classified by the Society of Automotive Engineers (SAE) according to their viscosity. Typical, monograde oils were classified according to the acronym SAE followed by a subordinate abbreviation, such as SAE30 or SAE5 W, which refer to viscosity values at specific engine operating temperatures. The comparison of viscosity‐temperature relationship of monograde oils have been studied in detail.[^22^, ^23^] The kinematic viscosity changes as a function of the temperature for the SAE30 (red line) and SAE5 W (blue line) are reported in Figure 2. SAE30, a high molecular weight base oil, has an adequate viscosity at high temperature but exhibits a very steep increase in viscosity at lower temperatures. Contrarily, lower molecular weight base oils, such as SAE5 W, generally have inadequate viscosity characteristics at high temperature.^[24]^ If a SAE5 W base oil is thickened with enough VM, such as polyethylene‐polypropylene random copolymer (PE‐*r*‐PP) (black line in Figure 2), the “multigrade” oil thus generated possesses the advantages of both lower molecular weight base oil (a less pronounced viscosity‐temperature slope) and higher molecular weight base oils (optimal viscosity at high temperature).[^25^, ^26^] The VM essentially acts as thickener for a base oil at high temperature maintaining the viscosity at low temperature optimal for the correct functionality of the motor engine.

According to the thermorheological properties conferred, VMs can be classified in viscosity index improvers and thickeners. A viscosity index improver (VII) mainly affects the viscosity of a base oil increasing it at high temperature but keeping it unchanged at low temperatures. In other words, VII keeps under control the rate of change in viscosity with temperature; a thickener increases the viscosity of the solution, but not necessarily increases the viscosity index.

As mentioned before, the *viscosity‐temperature relationship* is a critical property of VMs often quantified using the viscosity index (VI), a dimensionless variable that quantifies the rate of change of solution viscosity over a temperature range. The VI is defined by ASTM D2270 as the difference between the kinematic viscosity of a test oil at 40 °C (KV40) and the viscosity of a reference oil at 40 °C, divided by the difference between the viscosity of the test oil at 100 °C (KV100) and the viscosity of the reference oil at 100 °C.^[27]^ Ideally, VMs should also exhibit other fundamental characteristics such as good thickening in the working temperatures range of engines, preventing from friction and wear (*thickening efficiency*), as well as a shear resistance to mechanical stress (*shear stability*).

The *thickening efficiency* (TE) quantifies the amount of polymer required to achieve a desired viscosity in a lubricant formulation.^[23]^ As polymers are the largest cost item in lubricant formulations, this is a critical aspect for industrial lubricants.[^28^, ^29^] TE values between 0.5 to 4.0 are optimal.^[30]^

**Figure 3:**
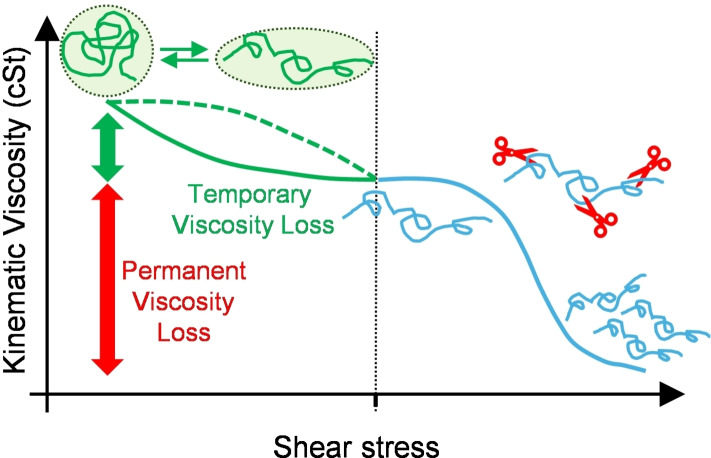
Schematic representation of *shear thinning* mechanism resulting in temporary and permanent viscosity losses.

The *shear stability* of VM polymers is a critical property that determines their performance: polymers can experience significant shear in lubricant applications, which can result in a phenomenon known as *shear thinning*, resulting in either temporary or permanent decreases in viscosity. Temporary viscosity losses occur when polymer modify their shape in response to increased shear rates. The permanent viscosity loss is due to the breaking of covalent bonds in the polymer chains, an irreversible process detrimental for sustained performances in lubrication (Figure 3). The behavior of different polymers in response to mechanical shear is not related to chemical composition, but rather it is a function of the polymer molecular weight and of the topology of the polymer chain.^[31]^

In general, lower molecular weight polymers tend to exhibit better shear stability, but they also have less thickening power. Polymer architecture plays an important role, with star‐shaped, graft‐, comb‐, and (hyper)branched structures generally providing improved shear stability compared to linear polymers with comparable molecular weights.^[32]^

**Figure 4:**
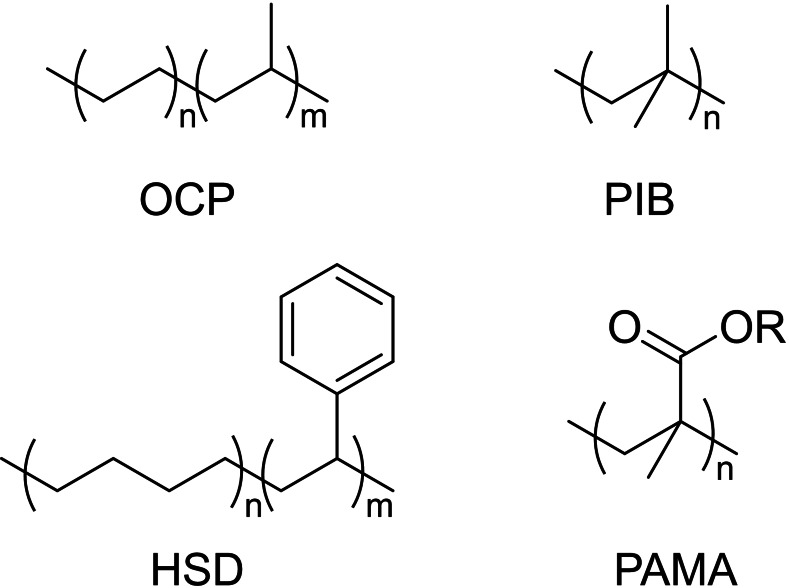
Chemical structures of typical VMs: OCPs, PIB, HSD and PAMA.

Polymers widely used industrially as VMs include poly(alkyl methacrylate) (PAMA), olefin copolymers (OCP), polyisobutylene (PIB), and hydrogenated styrene–diene (HSD), with molecular weights typically larger than 100 kDa. Their structures are shown in Figure 4.

**Figure 5:**
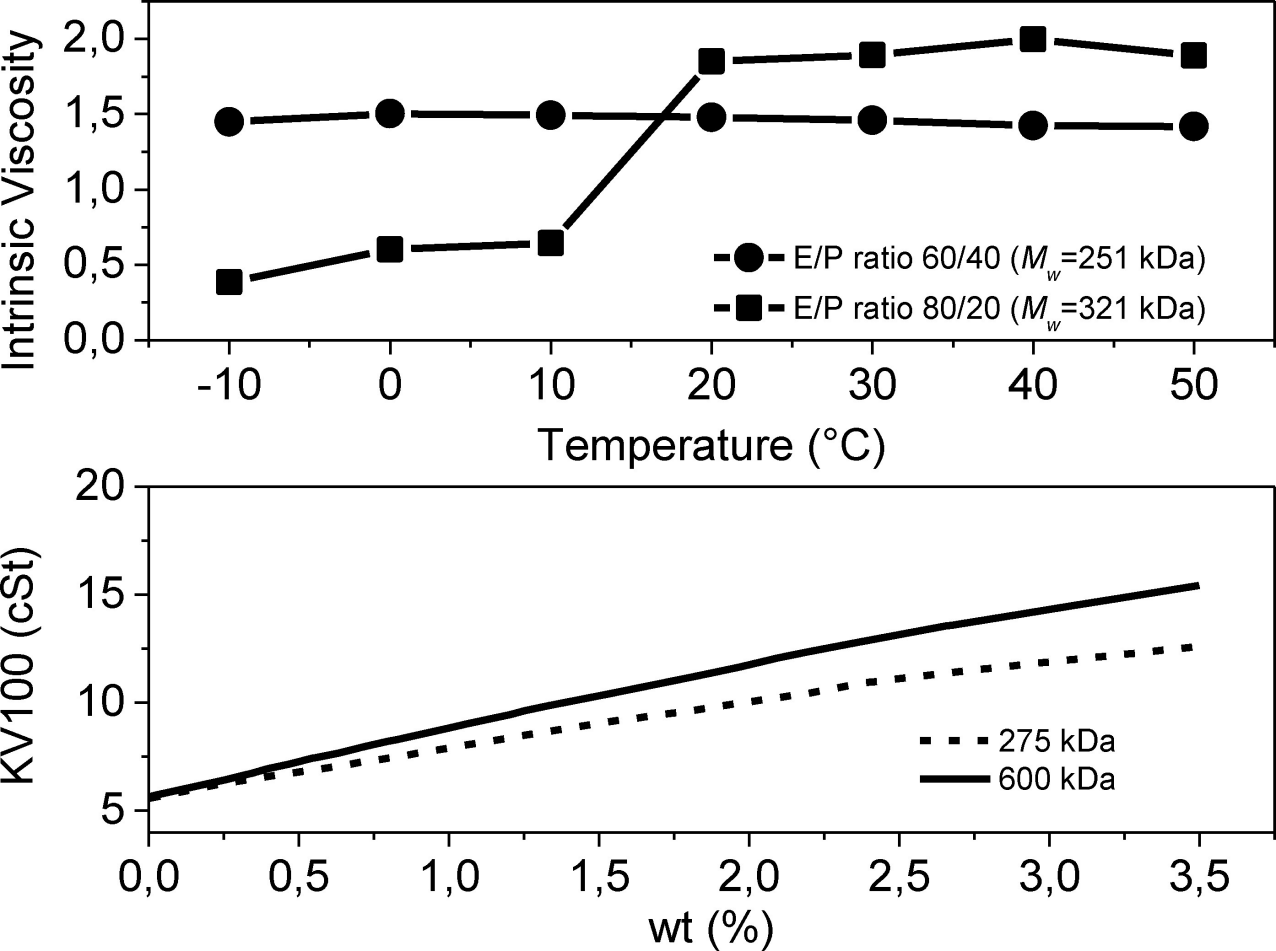
Top: variation of the intrinsic viscosity of different ethylene/propylene copolymer compositions with temperatures. Bottom: variation of KV100 with *M*_w_ for the same ethylene/propylene copolymer composition. Adapted with permission from ref.^[38]^ Copyright © 1990 John Wiley & Sons, Inc.

There are many different types of VMs that serve somewhat different purposes and perform their function through a variety of mechanisms. *OCPs and PIB* are used as VMs in a large proportion of the commercially available multigrade oils.^[33]^ Linear OCPs are prepared by solution polymerization of ethylene and propylene using Ziegler‐Natta catalysis,[^34^, ^35^, ^36^] whereas PIB is prepared industrially by cationic polymerization. The rheological characteristics of OCPs are strictly related to the ethylene/propylene ratio, molecular weight (*M*_w_), molecular weight distribution (*Ð*) and sequence distribution (random, gradient or block architectures). Indeed, while a high ethylene content improves TE, it decreases the solubility at low temperatures in base oils because of crystallization (Figure 5, top). On the other hand, a high propylene content decreases oxidative stability. The introduction of propylene into the backbone in the range of 40–60 w/w% gives sufficient solubility and stability in typical base oils. The *M*_w_ value is important to improve TE, the thermorheological properties (VI), as well as their shear stability (Figure 5, bottom).^[37]^ The molecular weight dispersity *Ð* is even more critical for setting previous parameters: broad *Ð* values, indicating significant amounts of low *M_w_* polymer chains, contribute to decrease the intrinsic viscosity.^[38]^ Large sections of ethylene blocks result in microcrystalline regions that have undesirable low solubility properties at low temperature, so that optimal OCPs presents a random distribution of monomers in the polymeric backbones.^[39]^ Gradient composition have also been explored and resulted in improved thickening power.^[40]^ Overall, OCPs are cost‐effective VMs suitable for applications in several formulations.

Another widely commercialized and well consolidated VMs chemistry is represented by *HSD polymers*.^[41]^ HSD copolymers are typically made using conjugated diene monomers, such as butadiene and isoprene, and alkenyl aromatics, with styrene as the most used. Anionic polymerization offers the best route to make different architectures (block, stars, telechelic, random) as a direct consequence of the living character of the process. Postpolymerization hydrogenation is done with heterogeneous catalysis (H_2_ with catalyst, such as Ni‐Raney) or with homogeneous methods using reactive diimides.[^41^, ^42^, ^43^]

The proportion of styrene and diene monomers adjusts the solubility and shear stability characteristics of the copolymer. The styrene block enhances the thermal, oxidative, and shear stability of the copolymer, but at the same time, it reduces the solubility. In fact, hydrogenated polystyrene–block–polyisoprene (PS‐*b*‐HPI) polymers with a >40 wt % in styrene composition have low solubility in base oils. The thermorheology of these VMs shows an abrupt viscosity change close to 100 °C. Below 80 °C the diblock copolymers are in a supramolecular star‐like aggregation, but when temperature rises, the styrene core swells as consequence of the increased solubility of styrene in oil, causing the disaggregation of micelles in unimolecular diblock copolymers.[^44^, ^45^]

The latest class of commercialized VMs chemistry is represented by *PAMA polymers*.^[46]^ PAMAs are efficient viscosity improvers due to their ability to increase viscosity at high temperatures while having minimal impact on viscosity at lower temperature. A commonly accepted mechanism that was first proposed by Selby relies on a reversible temperature‐induced coil expansion.^[47]^ The polarity of the ester groups is the root of this highly desirable thermorheological behaviors, which leads to a collapse of the macromolecular coil size at low temperature and to an hydrodynamic volume decrease. Compared to OCPs, PAMAs exhibit favorable low temperature properties, making them a popular choice for hydraulic and transmission fluids, but lately also for the new generation of automotive engine oils.

PAMAs backbones result chemically stable for lubricant applications. In fact, they are not susceptible to hydrolysis, thermal and oxidation reactions under normal conditions of use, there is little evidence that these reactions impact on the performances. The mechanical shear and thickening effects of PAMA are indisputably related to the molecular weight of the polymer backbone. PAMAs are not associative thickeners, unlike PS‐HSD. They do not experience viscosity losses through loss of molecular associations in high shear stress fields. Any loss of viscosity is solely due to the modification of molecular size caused by molecular shape distortion and breaking, which results in a lower hydrodynamic volume.

Commercial polymers are currently synthesized through free‐radical polymerization to give random copolymers with molecular weight ranging from 20 kDa to about 750 kDa. Typically, three types of alkyl methacrylates are used. The first class consists of short‐chain alkyl methacrylates, C1–C4 range. Short carbon chains amplify the polarity of ester groups. The second one contains C8–C13 sidechains and serves to improve the solubility of polymers in base oil solutions. Finally, the longer linear alkyl‐chain methacrylates (>C14) can interact with the base oil during its crystallization and thus provides pour point depressant behavior. The selected monomers are mixed in a specific ratio in order to provide an overall balance of the above‐mentioned characteristics.

### 2.2 The Mechanism of Action of VMs

The complex interactions and the supramolecular mechanisms between the VM materials and the base oil result in the formation of a wide variety of nano/micro/meso scale morphologies depending on the concentration of solutions.[^48^, ^49^] The interactions between the single chains of polymers and the molecules of the base oil are supramolecular in nature and their weaker nature gives rise to morphologies that are reversible. Morphology in self‐assembled objects is a result of free‐energy minimization,[^50^, ^51^] and such energies can be tuned by acting on the chemistry (composition and topology) through the modification of the strength and of the number of the weak interactions such as electrostatic bonds, hydrogen‐bonds, hydrophobic and *π‐π* stacking interactions. The most used instruments for measurement of the viscoelastic properties of polymer solutions are rheometers and viscometers, but numerous analytical techniques capable of yielding more advanced structural information in solution, such as hydrodynamic radius (*R_H_*) and gyration radius (*R_g_*), are gaining attention in this regard.[^54^, ^55^, ^56^, ^57^, ^58^, ^59^, ^60^]

**Figure 6:**
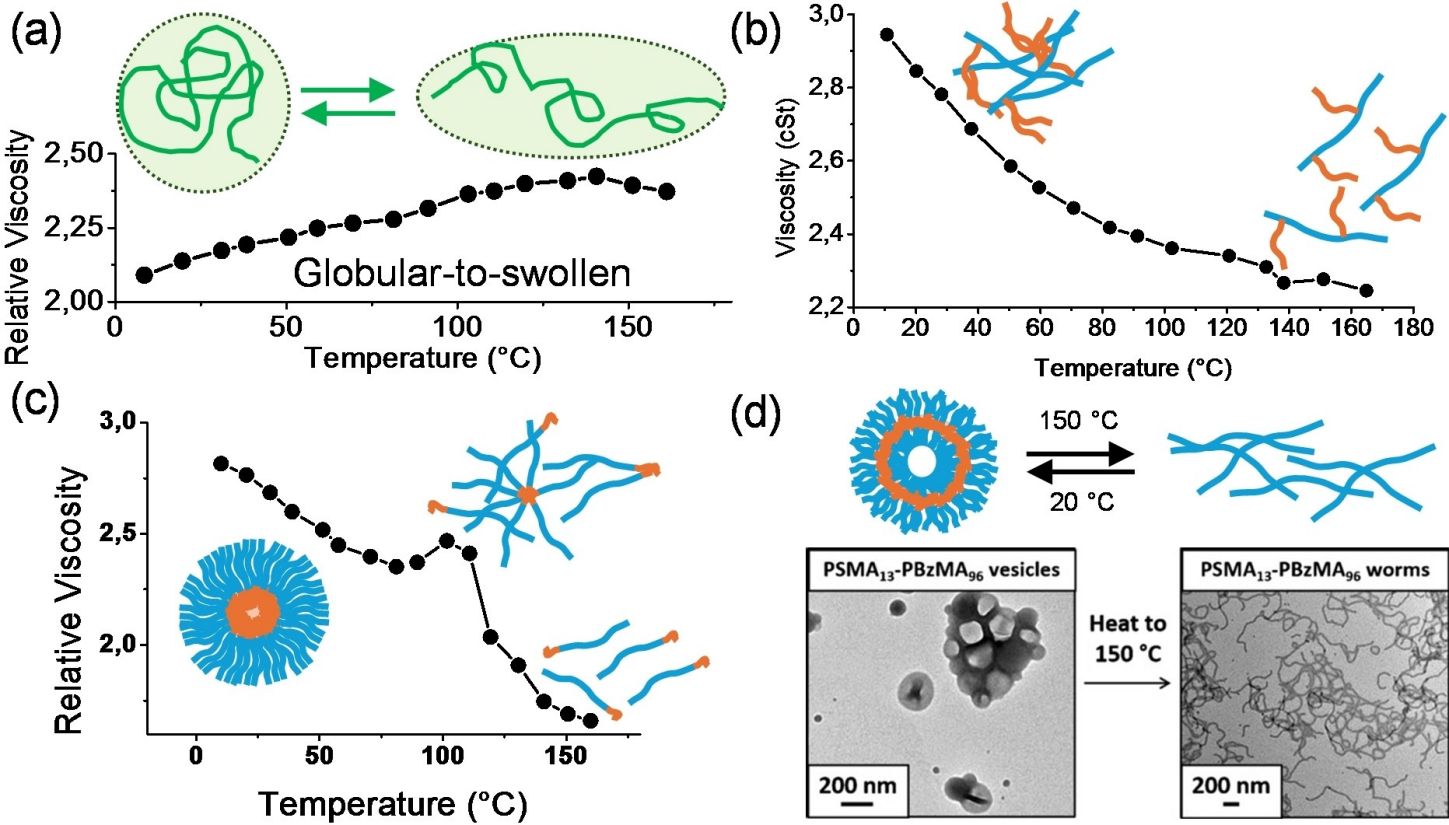
Viscosity‐temperature mechanisms reported in literature. (a) Selby mechanism for linear PAMA VMs.^[47]^ (b) Supramolecular dissociation proposed by Hillmyer for OPC‐*graft*‐PAMA VMs.^[63]^ (c) Micelles disaggregation proposed for HSD block‐copolymers.[^65^, ^66^] (d) Vesicles to worm‐like transition mechanism proposed by Armes.^[67]^ Partially adapted with permission from refs.[^47^, ^63^, ^65^, ^66^, ^67^] Copyright © 2018 American Chemical Society, 2023 Elsevier B.V.

The first mechanism proposed to explain the VI improvement of polymers was attributed to polymer coil swelling with increasing temperature, proposed by Selby for PAMA VMs and shown in Figure 6a.^[47]^ According to his description, a high‐molecular‐weight PAMA having poor solubility in base oil exhibits a compact conformation at low temperatures, but its polymer chain expands when temperature rises, and solubility as well. As a result, the VMs minimally affect the oil viscosity at a lower temperature and contribute more to viscosity at higher temperatures. The temperature‐induced transition from a random‐coil in good solvents to a collapsed globule in poor solvents has been the focus of many theoretical and experimental studies, mainly conducted on poly(styrene), measuring the polymer coil radius by dynamic light scattering (DLS) and small‐angle neutron scattering (SANS).[^54^, ^55^, ^56^, ^57^]

Recent studies have investigated the role of polymer architecture in enhancing the solution viscosity at higher temperatures for PAMA VMs. Helgeson and co‐workers confirmed that all PAMAs with different architectures (linear, branched and star‐shape) underwent temperature‐induced coil expansion, with the degree of chain swelling dependent on the polymer architecture.^[58]^ Viscosity‐temperature, DLS and SANS measurements experiments revealed that the degree of coil expansion is significantly lower for the star‐shaped additives when compared to the linear and randomly branched polymers, and underlined an extreme sensitivity in the interplay between polymer and oil.

The application of the globular‐to‐extended‐coil mechanism is not fully rationalizing the observed data also for other classes of VMs, such as OCPs and HSDs. Covitch and Trickett measured the variation of polymer coil size for three PAMAs and two OCPs VMs in deuterated dodecane solutions using SANS at 40 °C and 100 °C.^[59]^ The authors reported that only the VMs of PAMA show swelling with increasing temperature, and such swelling is influenced by the monomer composition, in agreement with the computational study by Martini *et al*. using molecular dynamics (MD) simulations.^[60]^ In the case of OCPs, the authors observed viscosity improving behavior unrelated to the typical increase in *R_g_*. Cosimbescu and co‐workers compared in hydrocarbon solvents two PAMAs (linear and star‐shaped), having polar backbone, and two OCPs (linear and hyperbranched), characterized by apolar backbones.^[61]^ Using DLS, SANS and MD simulations, the authors concluded that the observed thermoresponsive properties are not consistent with a globular‐to‐coil‐expansion mechanism, but that the viscosity changes with temperature are related to polymer–oil interactions and depend on the polarity of the polymer backbone.

If base oils are sufficiently good solvents over the operating temperature range, it is possible that the coil expansion, as measured by the change in *R_g_* and *R_H_* values, does not change significantly with temperature.^[62]^ Hillmyer *et al*. examined the microstructure of PAMA‐grafted polyolefins in dodecane using a variety of analytical techniques and found that both their *R*_g_ and *R*_h_ values decrease with increasing temperature.^[63]^ To rationalize this observation, the authors proposed that the polymers associate in mesh‐like supramolecular architectures at low temperatures due to an imbalance in solubility of the olefin backbone and methacrylate side chains (Figure 6b). At high temperatures, the polymer aggregates dissociate into single chains due to an increase in the solubility of the side chains, causing an overall decrease in the hydrodynamic volume of the polymer.

Chow *et al*. demonstrated, combining small‐angle X‐ray scattering (SAXS) and MD calculations, that the viscosity‐temperature relationship of poly(butyl methacrylate) (PBMA) is not correlated with the polymer swelling and emphasized the role of solvent polarity on the mechanism of action in dilute and semi dilute concentration regimes.^[64]^ The authors observed that intrinsic viscosity of PBMA decrease in polar solvents and increase in apolar solvents, and the two different behaviors are the result of two different mechanisms of action. Using SAXS the authors demonstrated that in polar solvents PBMA increases its *R_g_* values with increasing temperature, because at high temperature the solubility of the butyl chains increases, causing the swelling of the random‐coil conformation. In apolar solvent, PBMA decreases its *R_g_* values due to the supramolecular association of its polar polymeric backbone. Once again, the Selby mechanism cannot fully explain the experimental data.

The thermorheology of HDS VMs with block architecture is an interesting case that gives strength to the inapplicability of the Selby model for all polymers. In fact, HDS block polymers show an abrupt viscosity change close to 100 °C (Figure 6c), as well as well‐known associative polymers in water.[^65^, ^66^, ^67^] SANS measurements at different temperatures showed that below 80 °C the diblock copolymers are in a supramolecular star‐like aggregation, with the styrene block confined in the core of the micelles and the polyolefin arms outside the core acting as solubilizing groups. As the temperature approaches to 100 °C, the styrene core swells as consequence of the increased solubility of styrene in oil, causing the disaggregation of micelles in monomeric diblock copolymers.[^44^, ^45^] In 2017, Armes *et al*. demonstrate a VI improving mechanism, shown in Figure 6d, based on vesicle‐to‐worm transitions for linear PAMA block‐copolymer poly(lauryl methacrylate)‐block‐poly(benzyl methacrylate) (PLMA‐*b*‐PBzMA).^[68]^ Combining TEM, SAXS and NMR studies the authors demonstrated that PLMA‐*b*‐PBzMA in mineral oil at 25 °C self‐organize in polymersomes (vesicles), with the polymer chains organized into a lipid‐like bilayer membrane. The base oil‐soluble building blocks (PLMA) face outwards, interacting with the apolar solution, while the polar chains (PBzMA) lie in the core of the bilayer, interacting with each other through *π‐π* stacking interactions. The rise in temperature leads to a thinning of the bilayer due to increased solvation of the benzyl groups, and at 135 °C the vesicles collapse and the vesicle to worm‐like transition occur. In follow up studies, Zhang and co‐workers have effectively demonstrated the use of (PLMA‐*b*‐PBzMA) as viscosity modifiers for a monograde oil.^[69]^

### 2.3 Innovative Polymer Chemistry for VMs

In this section we will analyze the different polymeric architectures used as VMs and how their performance is affected. A few reviews have been reported over the past 30 years on this subject.[^23^, ^26^, ^33^, ^41^, ^46^, ^70^]

#### 2.3.1 Olefin Copolymers

Architecture modifications for OCPs continue to be developed to enhance VMs performance. Zhu *et al*. synthesized a range of hyperbranched polyethylenes with controllable chain topologies, high branching density, molecular weight (up to 161 kDa) and moderate polydispersity (2.1 – 2.7).[^71^, ^72^, ^73^] The polymers showed extremely high shear stability and good thermorheological properties at low temperature with no crystalline structure at high *M_w_* values. More recently, Cosimbescu *et al*. prepared hyperbranched OCPs with ethylene and methyl 10‐undecenoate (<10 mol %) to increase polarity and encourage polymer‐surface interactions.^[74]^

**Figure 7:**
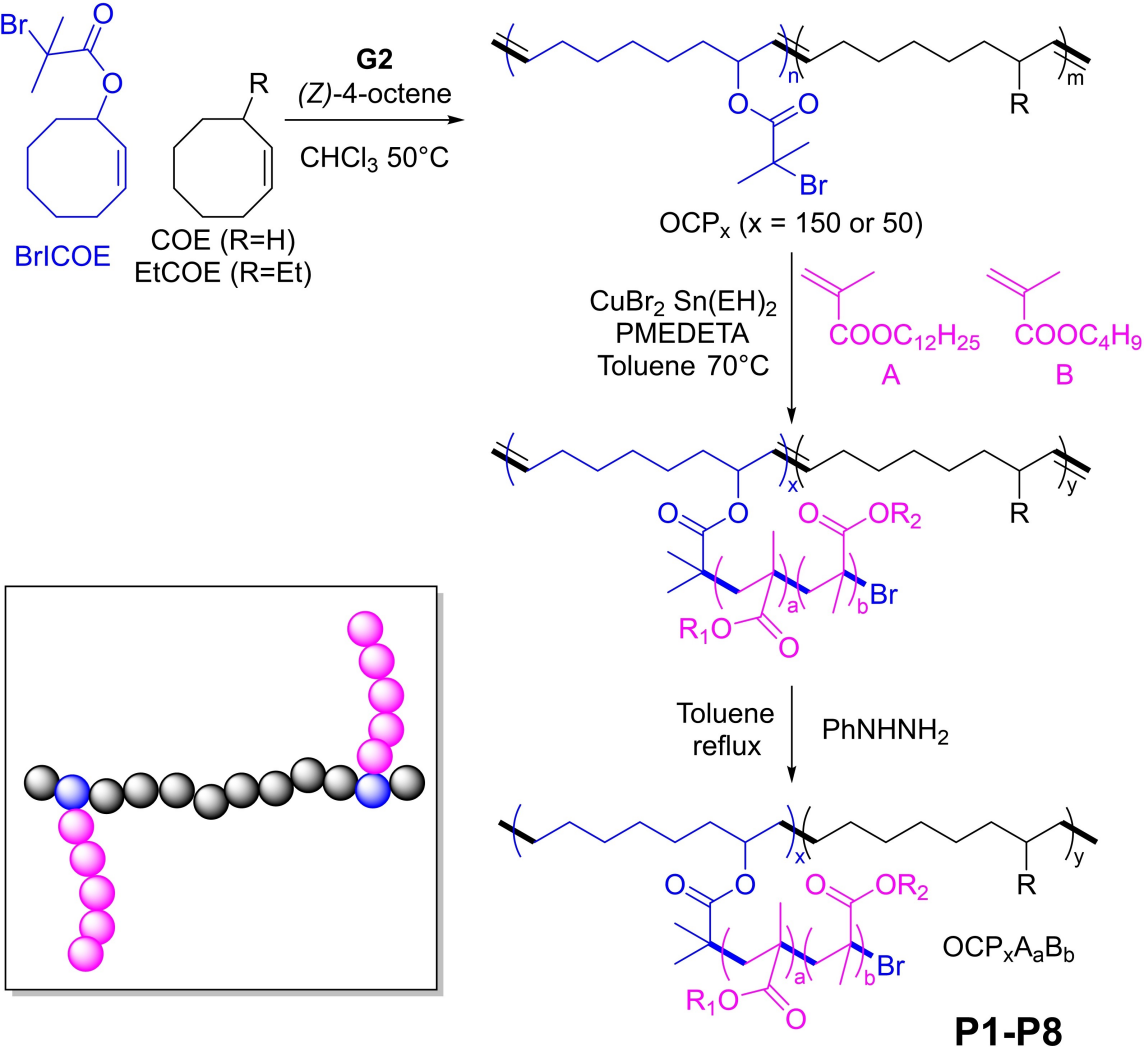
Synthesis of grafted copolymer P1–P8 by Hillmyer *et al*.^[63]^

Generally, OCPs act more as thickeners rather than VII. This observation prompted by Hillmyer and co‐workers to combine OCPs and PAMAs in a graft‐copolymer architecture,^[63]^ to take advantage of both classes of polymers: OCPs are thickeners with a low VI values at high‐temperatures, while PAMAs offers improved VI properties over a wide temperature range and interfere with the formation of wax crystals in the oil keeping relatively lower oil viscosity at low‐temperatures. The authors prepared a series of graft copolymers with different molecular compositions based on polyolefin backbones and PAMA side chains (Figure 7). Polyolefin backbones were synthesized by ring opening metathesis (co)polymerization (ROMP) of cis‐cyclooctene (COE), 3‐ethyl COE (3EtCOE), and α‐bromoisobutyrate functionalized cis‐cyclooctene (BrICOE).

Two ATRP macroinitiators were prepared containing 8 % BrICOE (corresponding to an average of one side chain per 100 carbon atoms of the polyolefin backbone), and 92 % of comonomer COE, differing as for the overall molecular weight and monomer composition (OCP_50_ present a *M*_n_ value of 50 kDa and COE/EtCOE ratio of 100:0, while OCP_150_, present a *M*_n_ value of 150 kDa and COE/EtCOE ratio of 25 : 75). The unsaturated polyolefin precursors were used in a “grafting‐from” reaction of the methacrylate monomers through an activator‐regenerated‐electron‐transfer (ARGET) atom‐transfer‐radical‐polymerization (ATRP), prior to chemical hydrogenation. Two alkyl methacrylate monomers were selected to prepare the side chains by statistical copolymerization: lauryl methacrylate (A) and butyl methacrylate (B). The molecular characteristics and thermorheological properties of synthesized VMs **P1–P8** are reported in Table 1.

**Table 1:** Thermorheological properties in base oil of polymers P1–P8.

Polymers	Structure	*M*_n_ (kDa)	*Ð*	wt %	KV40	KV100	VI
base oil	‐	‐	‐	‐	22	4.4	109
P1	OCP_150_A_210_B_0_	215	2.2	1.3	41	8.4	187
P2	OCP_150_A_290_B_0_	300	2.1	1.2	40	8.4	193
P3	OCP_150_A_760_B_0_	750	2.4	0.8	38	8.4	207
P4	OCP_150_A_60_B_40_	610	3.6	1.0	37	8.4	214
P5	OCP_150_A_40_B_60_	600	2.7	0.7	34	8.1	225
P6	OCP_50_A_260_B_0_	290	2.1	1.3	39	8.3	196
P7	OCP_50_A_60_B_40_	260	2.1	1.0	39	8.2	192
P8	OCP_50_A_40_B_60_	660	2.2	0.8	34	8.2	229

The authors studied the effect of the PAMA side chain length on VM performance in **P1–P3**, with OCP_150_ as backbone. The wt % required to achieve a KV100 value close to 8 centistokes (cSt) decreases when the PAMA side chain length increases, because the hydrodynamic volume become larger with larger *M*_n_ values. Two polymers with varying fractions of A and B monomers were prepared (**P4** and **P5**) to explore the effect of the alkyl chains.

The KV40 values decreases as the fraction of B increases, while KV100 values remain almost constant. The series **P6–P8**, with a polyolefin backbone without 3EtCOE comonomer, was synthesized (OCP_50_) to explore the influence of branching in the backbone. Without ethyl branches, the polymers showed a higher propensity to crystallize than those synthesized from CE_150_ macroinitiators (**P3–P6**). Size measurements by DLS, SLS, and PFG‐NMR indicated that coil expansion upon increasing temperature is not the mechanism for the viscosity improving behavior of these graft‐copolymers in oil. Instead, the SANS results suggest that the polymers associate in mesh‐like supramolecular architecture at low temperatures due to an imbalance in solubility of the olefin backbone and methacrylate side chains. At elevated temperatures, the polymer aggregates dissociate into single chains due to an increase in the solubility of the side chains, causing an overall decrease in the hydrodynamic volume of the polymer.

#### 2.3.2 Hydrogenated Styrene‐Diene Copolymers

The applicability of lining polymerization techniques in HSD copolymers has given access to a series of innovative architectures to be tested as VMs. Olson and Handlin, for example, synthesized triblock copolymers composed of hydrogenated isoprene and polystyrene block (HPI‐*b*‐PS‐*b*‐HPI) that exhibit good balance of high‐temperature viscosity, TE and shear stability.^[75]^

**Figure 8:**
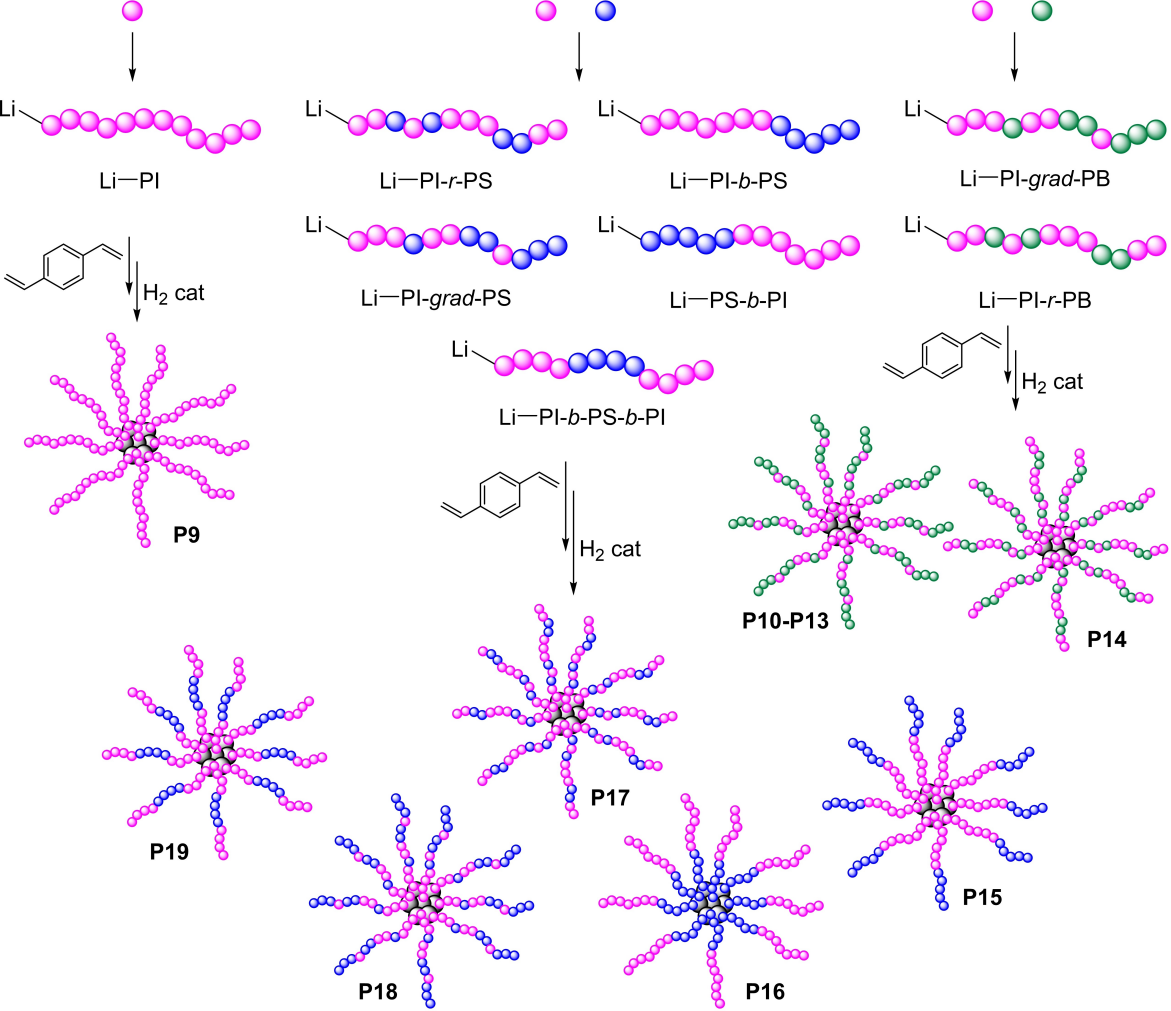
Star‐copolymer via anionic random/block‐copolymerization of styrene, 1,4‐butadiene and isoprene monomers (P9–P19).[^76^, ^77^, ^78^] Purple dots: isoprene (I); blue dots: styrene (S); green dots: butadiene (B)

In addition to linear HSD random or block copolymers, star polymers also find application in lubrications. Star‐like, random/block copolymers are prepared by first forming linear random/block polymers having active Li atoms at one end of the polymer chain. Which are then reacted with compounds acting as crosslinkers, having at least two functional groups able to react with Li‐terminated polymers (Figure 8): a cross‐linked nucleus is formed, with the polymer arms dangling out into the solution. Preferred crosslinkers are divinyl or trivinyl aromatic compounds or aliphatic or aromatic diisocyanates. The number of arms varies typically in the 10–20 range. Finally, the star‐copolymer is hydrogenated. These structures are very stable thanks to the internal stress distribution on all arms. They are chemically cross‐linked and do not dissociate at high temperatures.

Fetter and Eckert synthesized star‐polymer **P9–P18** having arms composed by hydrogenated homopolymers and gradient copolymers of conjugated diene and styrene reported in Table 2 and shown in Figure 8.[^76^, ^77^]

**Table 2:** Thermorheological properties in base oil of HSD polymers P9–P19.

Polymers	Structure	*M_w_* (kDa)	wt %	KV40	KV100	VI
P9	(PI)_star_	520	1.5	133.2	19.0	172
P10	(PI‐*grad*‐PB)_star_	344	1.9	133.4	19.1	173
P11	(PI‐*grad*‐PB)_star_	421	1.6	131.5	18.8	171
P12	(PI‐*grad*‐PB)_star_	489	1.4	132.3	18.9	172
P13	(PI‐*grad*‐PB)_star_	557	1.3	138.2	19.6	173
P14	(PI‐*r*‐PB)_star_	643	1.5	135.7	19.8	177
P15	(PI‐*b*‐PS)_star_	504	1.5	134.5	19.1	171
P16	(PS‐*b*‐PI)_star_	499	1.7	127.2	18.4	172
P17	(PI‐*r*‐PS)_star_	451	1.8	135.0	19.2	172
P18	(PI‐*grad*‐PS)_star_	489	1.7	129.7	18.8	174
P19	(PI‐*b*‐PS‐*b*‐PI)_star_	585	1.5	129.1	18.7	173

The long, unsubstituted linear alkyl sequences, both in homopolymers and in copolymers, present the same low‐temperature performance problems observed in OCPs with high ethylene content. To provide an improvement in TE, Rhodes *et al*. developed star polymers comprising triblock copolymer arms composed by HPI‐HPB‐HPI, such as **P19**.^[78]^ The hydrogenated polybutadiene block provided an increased ethylene content, which improves TE while leaving the VI essentially unchanged. The authors suggested that a more proximity of HPB segment to the nucleus led to a beneficial effect on low‐temperature properties. In fact, such polymers were found to provide improved low‐temperature properties relative to the gradient arm polymers of Eckert.

#### 2.3.3 Poly(alkyl methacrylate)s

The evolution of efficient processes for controlled‐radical polymerization (CRP) in the 1990s has led to the development of new architectures and copolymerization, as well as the development of VMs with narrower molecular weight distributions. For instance, commercial PAMA VMs have been synthesized successfully through reversible addition−fragmentation chain‐transfer polymerization (RAFT), atom‐transfer radical polymerization (ATRP) and nitroxide‐mediated polymerization (NMP) from alkyl methacrylate monomers bearing alkyl groups of different chain lengths and polarity.[^79^, ^80^, ^81^, ^82^] Novel trends in PAMA VMs are focused on the development of novel architectures and materials able to better target functional properties.

Hybridization of VM chemistry is an increasingly used strategy towards materials with improved properties. Müller, Eisenberg and Stöhr combined PAMA and OCP chemistries in well‐defined grafted copolymers.^[83]^

**Figure 9:**
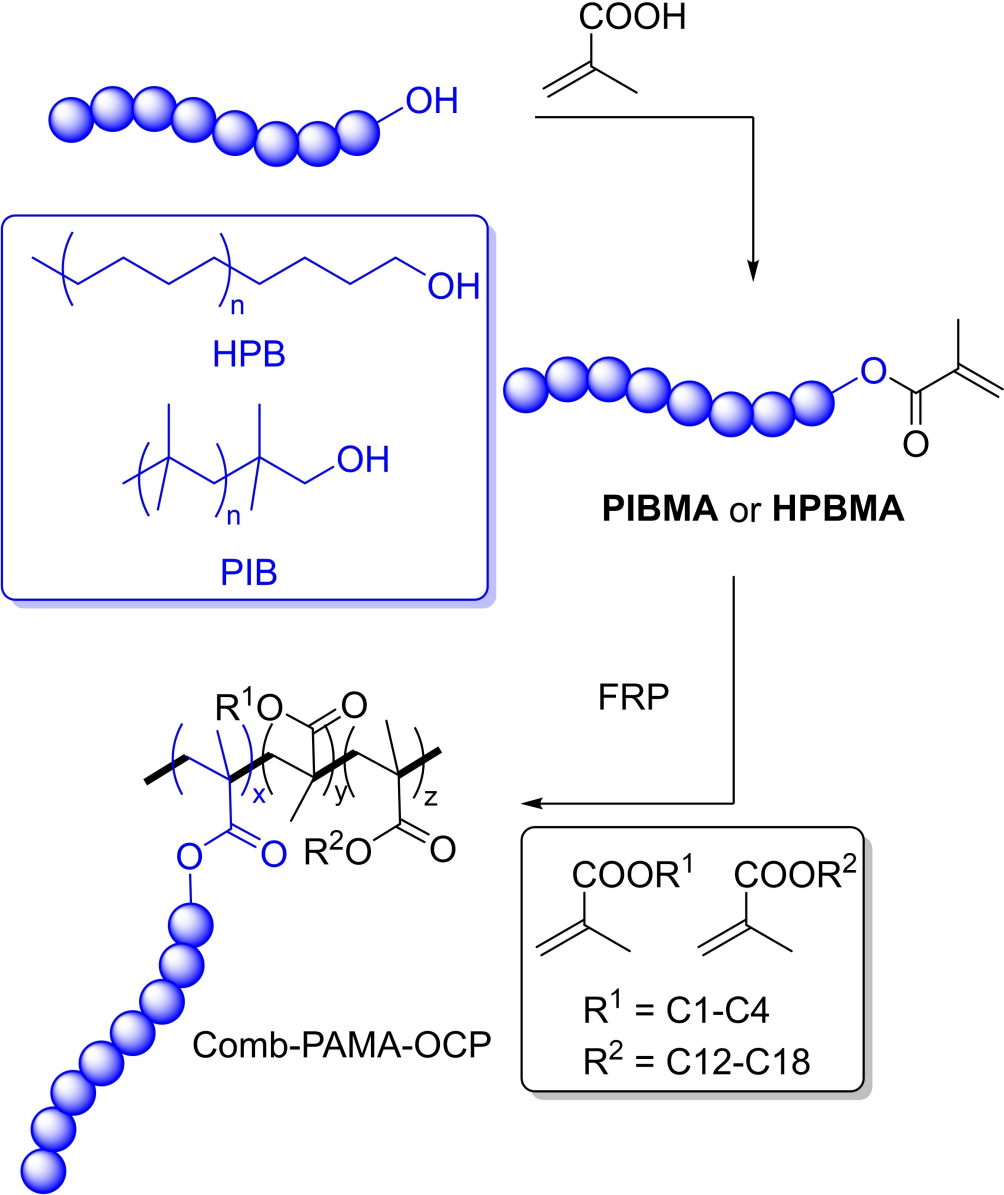
Synthesis of comb‐PAMA grafted with PIB and HPB as OCPs.^[83]^

As shown in Figure 9, the key raw materials are polyolefin macroalcohols based on poly(isobutene) (PIB) or hydrogenated poly(butadiene) (HPB) with molecular weights in the range 1.0–10.0 kDa. The authors synthesized a library of comb‐PAMA‐OCPs in two synthetic steps: an esterification reaction with methacrylic acid to give the macromonomers PIBMA and HPBMA, followed by their free‐radical polymerization with a mixture of C1–C4 and C12–18 methacrylate monomers. Comb‐PAMA grafted with PIB and HPB yielded multigrade oils with competitive TEs, VIs, and shear stability properties relative to OCPs and PAMAs.

**Figure 10:**
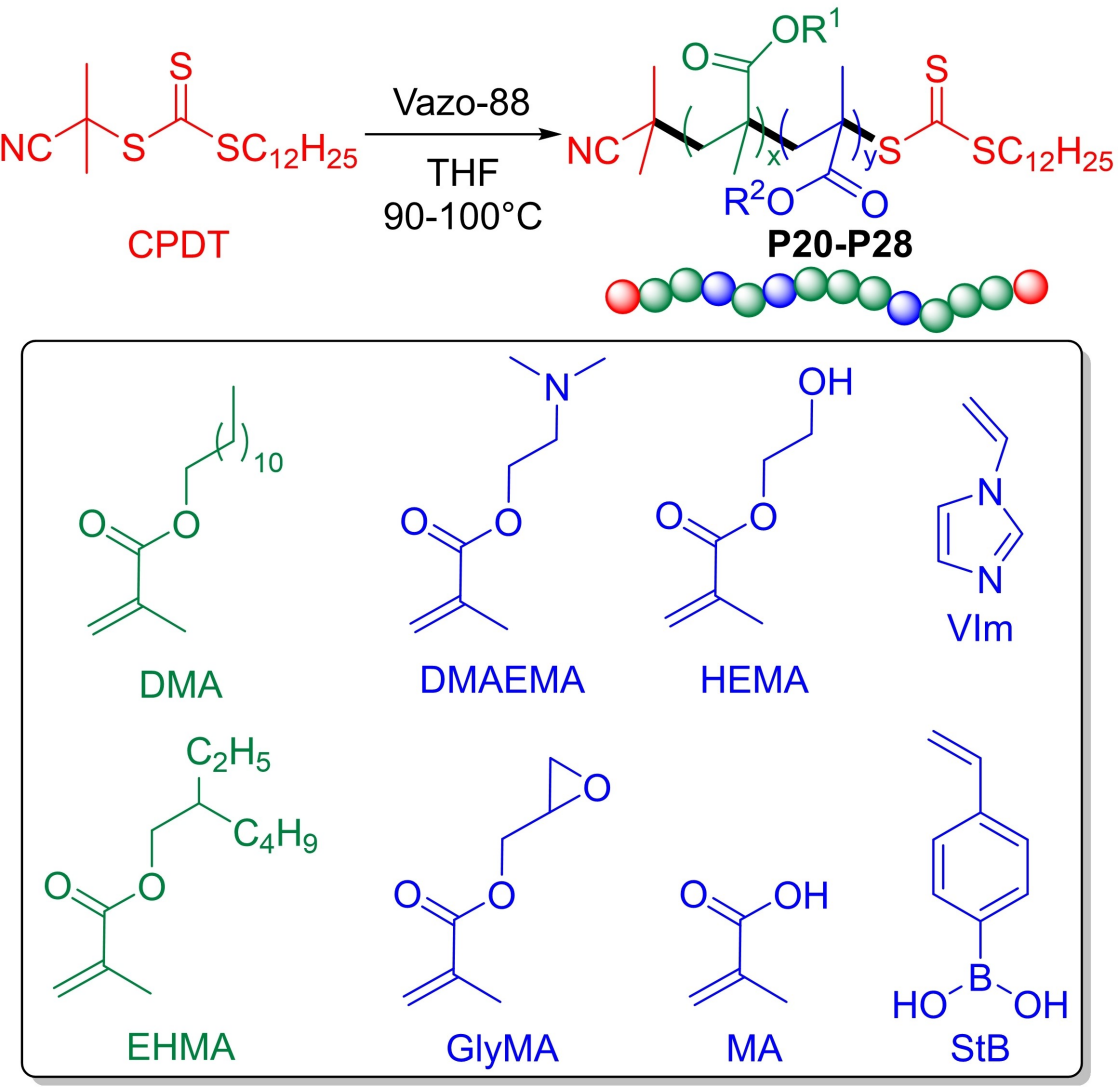
(a) Synthesis of linear PAMA polymers P20‐P28 via RAFT polymerization.^[85]^

Smart VMs able to serves at same time as VMs and wear prevention or friction modifiers,^[84]^ are object of great attention in last years. Cosimbescu *et al*. reported the synthesis of novel linear PAMA **P20–P28**, reported in Table 3, exhibiting the dual functionality of VI improvers and wear prevention simply introducing polar groups able to interact with metal surfaces.^[85]^ The authors synthesized a series of homopolymers and copolymers with molecular weights targeted around 150 kDa via RAFT polymerization using CPDT as chain‐transfer agent (CTA) and Vazo‐88 as a radical initiator (Figure 10).

**Table 3:** Thermorheological properties in base oil of HSD polymers P20–P28.

Polymers	Structure	*M_n_* (kDa)	Ð	wt %	KV40	KV100	VI
base oil	70 %100 R/30 %220 R	‐	‐	‐	24.3	4.80	116
P20	PEHMA_750_	151	3.10	2.0	30.0	6.38	171
P21	PDMA_750_	126	1.77	2.0	31.1	6.44	165
P22	PDMA_675_‐*r*‐PMA_75_	137	2.39	2.0	27.4	5.52	144
P23	PDMA_625_‐*r*‐PGlyMA_125_	171	2.29	2.0	31.4	6.90	189
P24	PDMA_625_‐*r*‐PDMAEMA_125_	156	2.90	2.0	35.3	7.10	168
P25	PDMA_625_‐*r*‐PHEMA_125_	124	1.99	2.0	26.5	5.75	167
P26	PDMA_625_‐*r*‐PVIm_125_	59.6	3.10	2.0	31.6	6.63	172
P27	PDMA_250_‐*r*‐PVIm_50_	45.6	2.37	2.0	28.6	6.00	162
P28	PDMA‐*r*‐PStB	150	2.59	2.0	34.5	6.95	167
P29	PSMA‐*r‐*PAAEMA_18_	203	1.20	2.5	58.6	‐	‐
NP29	PSMA‐*r‐*PAAEMA_18_	233	1.30	2.4	54.3	7.80	126
P30	PSMA‐*r‐*PAAEMA_27_	138	1.10	2.6	49.7	‐	132
NP30	PSMA‐*r‐*PAAEMA_27_	145	1.10	2.4	49.9	7.9	‐114
P31	PSMA‐*r‐*PAAEMA_21_	205	1.30	1.0	28.0	4.6	103
NP31	PSMA‐*r‐*PAAEMA_21_	264	1.30	1.0	27.9	4.6	104

All copolymers were obtained combining a non‐polar monomer, dodecyl methacrylate (DMA) or 2‐ethylhexyl methacrylate (EHMA), with a series of polar monomers: vinyl imidazole (VIm), 2‐hydroxyethyl methacrylate (HEMA), methacrylic acid (MA), glycidyl methacrylate (GlyMA), dimethylaminomethyl methacrylate (DMAEMA). The authors synthesized also copolymers of DMA with 4,4,5,5‐tetramethyl‐2‐(4‐vinylphenyl)‐1,3,2‐dioxaborolane (StB) via free‐radical polymerization. All homopolymers were also prepared for comparison. Thermorheological properties are reported in Table 3.

All polymers had higher thickening efficiencies than the benchmark at 2 wt %, resulting in higher KV100 s, except for **P22**, **P25** and **P27**. Generally, the VIs track well with the *M*_w_, exception of **P26**, which has an anomalous, low *M*_w_ value of 59.5 kDa but one of the highest VI value. The coefficient of friction of all polymers was at the very least comparable to the commercial benchmark, except again for **P26**, which showed a significant friction reduction of 25 %. Overall, the authors demonstrated that their multifunctional polymers not only provide VIs and shear stability comparable to commercial materials but also with lower friction and superior wear protection.

**Figure 11:**
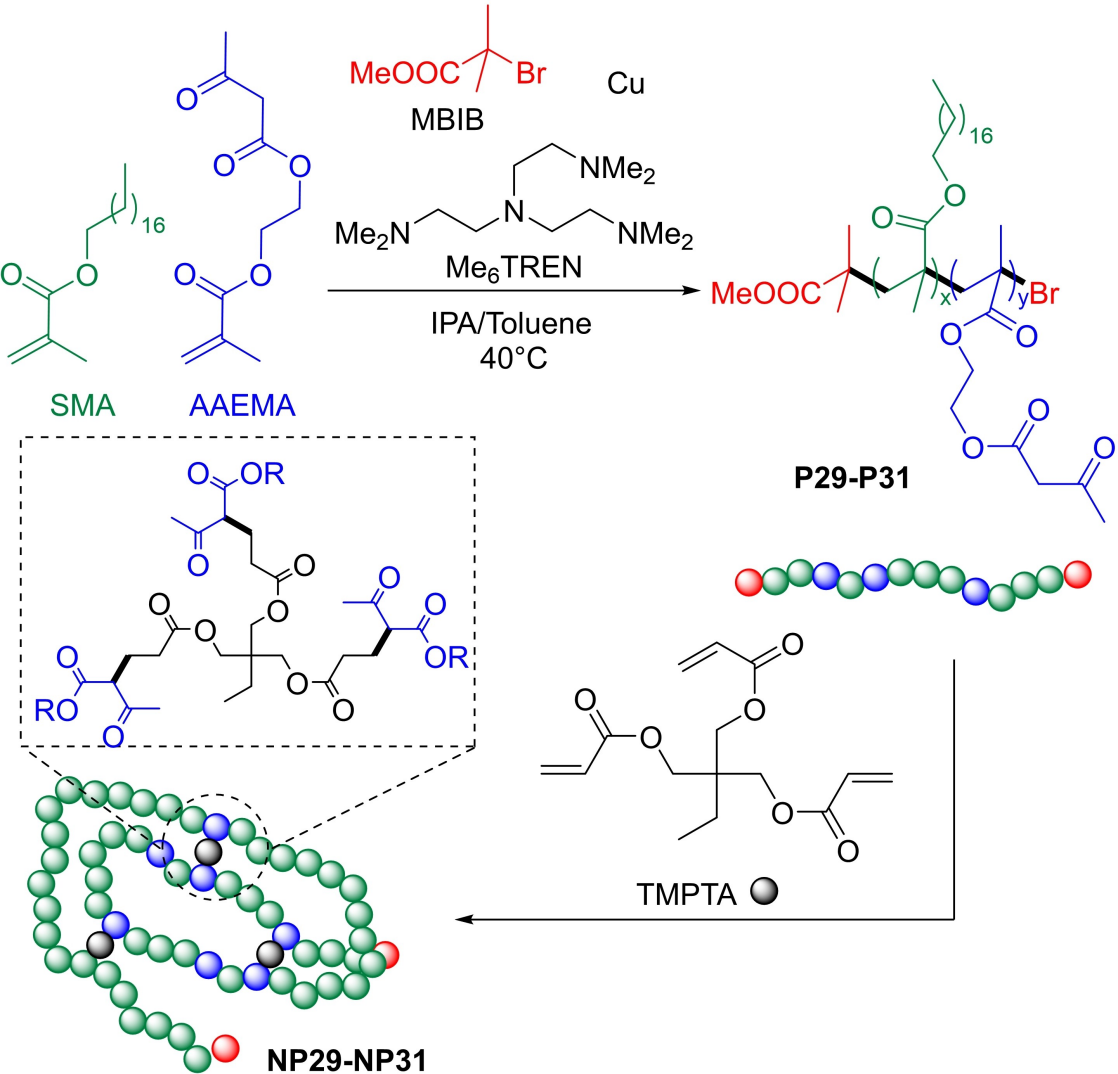
Synthesis of linear PAMA SCPNs NP29‐NP31.^[86]^

Diesendruck *et al*. reported an interesting innovation in linear PAMA chemistry that make use of single‐chain polymer nanoparticles (SCPNs) technology.^[86]^ SCPNs are polymeric nanoparticles prepared from single parental polymer chains through intrachain folding in dilute solutions. The collapse of polymer backbones to form nano‐objects is driven by covalent or noncovalent intramolecular cross‐linking interactions of suitable complementary functional groups, mimicking the folding of proteins.[^87^, ^88^, ^89^] The authors synthesized a series of parental copolymers **P29–P31** and corresponding nanoparticles (NPs) **NP29–NP31** with molecular weights between 138 – 205 kDa via ATRP polymerization using MBIB as initiator and Cu/Me_6_TREN as catalyst (see Table 3 and Figure 11), in presence of SMA and AAEMA as monomers. SMA provides the required lipophilicity, while AAEMA is a polar H‐bond forming monomer, which allows cross‐linking reaction with the Michael acceptor trimethylolpropane‐tetra acrylate (TMPTA). The parental copolymer **P29–P31** were covalently folded into corresponding NPs (**NP29–NP31**) in four days with the concentration in parental polymers of 1.8 g L^−1^. Rheological studies demonstrate that linear polymers are slightly better thickeners compared to their NPs, and comparable viscosity index improvers (see Table 3). Permanent viscosity loss (PVL) measurements showed superior resilience to shear of the SCPN VMs with respect to the linear polymers.

**Figure 12:**
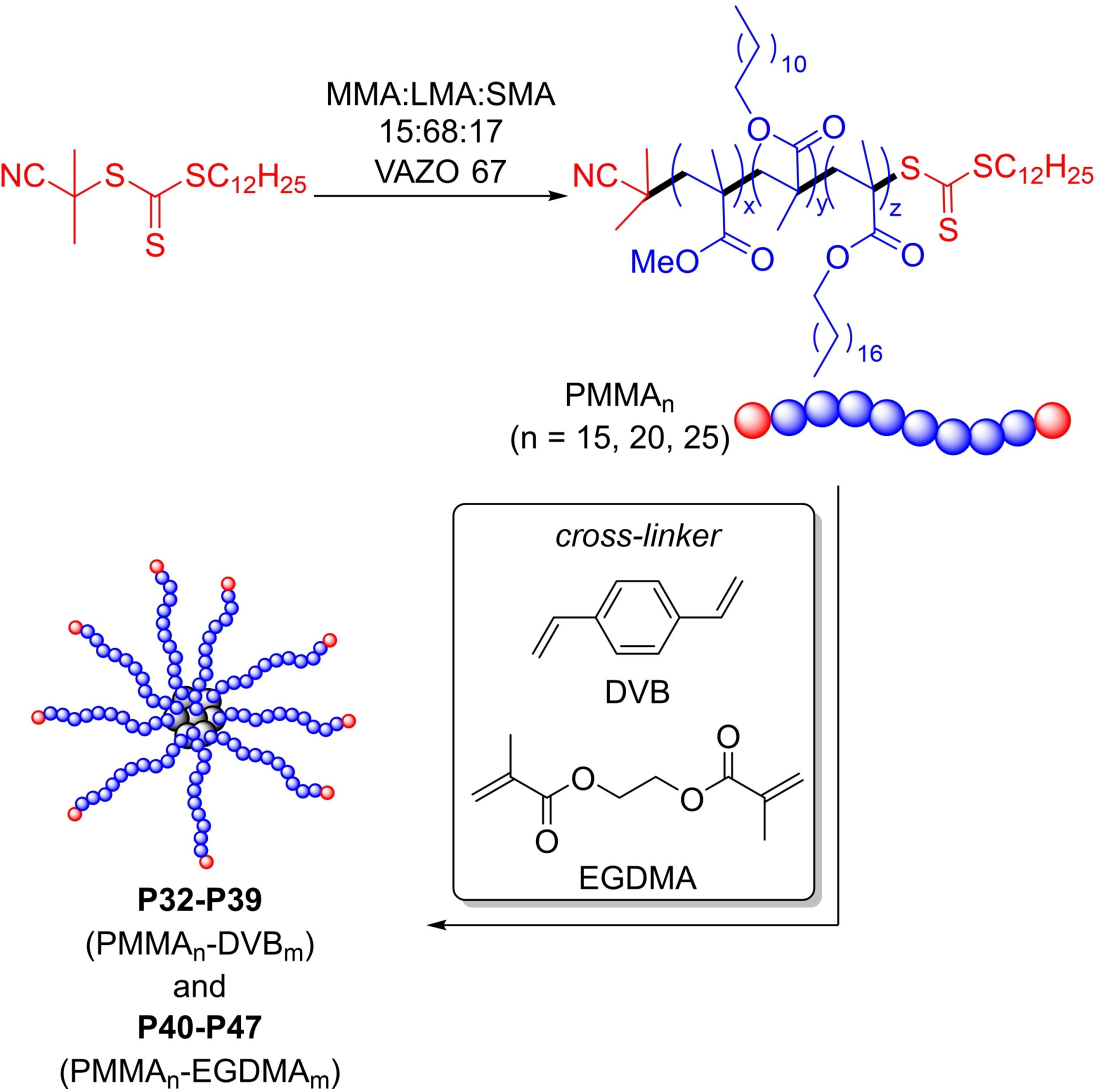
Strategies for the synthesis of star‐polymers. (a) “arm‐first” approach for the synthesis of polymers P32–P47.^[92]^

Due to their high VIs and good mechanical shear degradation resiliency, star‐shaped PAMAs are an ideal lubricant additive. Pioneers in PAMA‐based star‐shaped VMs were Lubrizol team that demonstrated the resiliency to strong mechanical forces for this architecture.[^90^, ^91^] Such polymers were obtained with an “arm‐first“ synthetic strategy reported in Figure 12, consisting in the synthesis of PAMA‐based arms followed by they linking to form the core of the final star‐shape architecture using a cross‐linking agents. These structures are generally referred to as core cross‐linked star (CCS) polymers.

Recently, Sparnacci *et al*. reported the synthesis of CCS polymer series **P32–P47** as VII for lubricants using the “arm‐first” strategy.^[92]^ The synthesis of such star‐polymers is reported in Figure 11a, while their properties are summarized in Table 4.

**Table 4:** Thermorheological behaviour of star‐polymers P32–P56.

Polymers	Structure	*M_w_* (kDa)	*Ð*	N_arms_	wt %	KV40	KV100	VI
Benchmark	HiTEC 629	‐	‐		3.1	47.2	8.1	145
P32	PAMA_25_‐DVB_5_	130	1.09	4	3.75	46.1	8.4	159
P33	PAMA_25_‐DVB_10_	163	1.10	5	3.5	44.7	8.0	154
P34	PAMA_25_‐DVB_15_	206	1.11	6	2.8	45.5	8.2	155
P45	PAMA_25_‐DVB_20_	314	1.26	10	2.2	45.2	8.2	156
P36	PAMA_20_‐DVB_5_	174	1.10	5	3.0	46.1	8.0	148
P37	PAMA_20_‐DVB_10_	200	1.19	7	2.8	46.3	8.1	156
P38	PAMA_20_‐DVB_15_	298	1.27	8	2.5	45.7	7.9	146
P39	PAMA_20_‐DVB_20_	359	1.35	13	2.1	46.7	8.23	152
P40	PAMA_20_‐EGDMA_5_	159	1.09	6	3.5	45.2	7.8	144
P41	PAMA_20_‐EGDMA_10_	245	1.11	8	3.0	44.9	7.8	145
P42	PAMA_20_‐EGDMA_15_	359	1.15	10	2.8	46.1	8.2	151
P43	PAMA_20_‐EGDMA_20_	420	1.20	11	2.7	46.4	5.2	154
P44	PAMA_15_‐EGDMA_5_	157	1.11	7	3.5	44.3	7.7	144
P45	PAMA_15_‐EGDMA_10_	241	1.13	10	3.2	44.2	7.7	144
P46	PAMA_15_‐EGDMA_15_	308	1.15	11	3.0	44.0	7.7	144
P47	PAMA_15_‐EGDMA_20_	370	1.25	13	3.0	44.4	7.7	144
P48	C_3_‐PLMA_117_	245	2.7	3	2.0	28.3	6.4	188
P49	C_3_‐PLMA_168_	395	2.8	3	2.0	30.4	7.0	204
P50	C_3_‐PSMA_140_	112	1.5	3	2.0	24.4	5.4	165
P51	C_4_‐PLMA_173_	159	1.4	4	2.0	26.6	6.1	187
P52	C_6_‐PLMA_180_	265	1.7	6	2.0	26.9	6.2	190
P53	C_3_‐(PLMA‐*r*‐EGMA)_133_	185	3.6	3	2.0	25.5	5.8	185
P54	C_3_‐(PLMA‐*r*‐ST)_408_	154	1.8	3	2.0	23.7	5.3	163
P55	C_3_‐(PLMA‐*r*‐GlyMA)_n_	327	2.1	3	2.0	22.7	5.0	150
P56	C_3_‐(PLMA‐*r*‐MMA)_460_	220	2.6	3	2.0	23.8	5.9	208

PAMA arms were obtained by RAFT polymerization starting from a mixture of MMA, LMA, and SMA with 15 : 68 : 17 ratio, keeping the arms composition constant in all materials, targeting the three different molecular weights values of 15.0, 20.0 and 25.0 kDa (PAMA_15_, PAMA_20_ and PAMA_25_, respectively). CCS copolymers **P32–P47** were then prepared using two cross‐linkers, DVB and EGDMA, varying the molar ratio of cross‐linker/PAMAn to obtain CCS with different number or arms. All star‐polymers presented a *M*_w_ ranging from 130.0–420.0 kDa and number of arms ranging from 4 to 13. Independently by the cross‐linker used, when starting from arms with a lower *M*_w_ values CCS copolymers with a higher number of arms are obtained. The VMs characteristics were studied in lubricant formulation prepared adjusting the wt % of polymers to obtain KV40 values in a range of 45–50 cSt. For each CCS polymers, the authors observed an increase of TE values with an increasing in *M_w_* of both star‐copolymers and the single arms, as consequence of an increase of intermolecular chain entanglement. These lubricant formulations exhibit good shear stability when reducing the *M_w_* of the star copolymer. A good compromise between VI improvement and capability to resist chain breaking reactions is obtained for CCS **P44–P47** copolymers with lower *M_w_* value for the PMMA arms. All samples were employed without any purification step in the preparation of model lubricant formulations.

“Arm‐first” approaches were also reported using an ATRP approach instead of RAFT by Tang *et al. via* ATRP protocol using the same crosslinkers (DVB, EGDMA) used previously.^[93]^ The crosslinkers were, differently from the two step synthetic approach illustrated in Figure 12, introduced in the reaction mixture at the same time of the monomers, giving rise to irregularly branched polymers, which were characterized as VMs.

**Figure 13:**
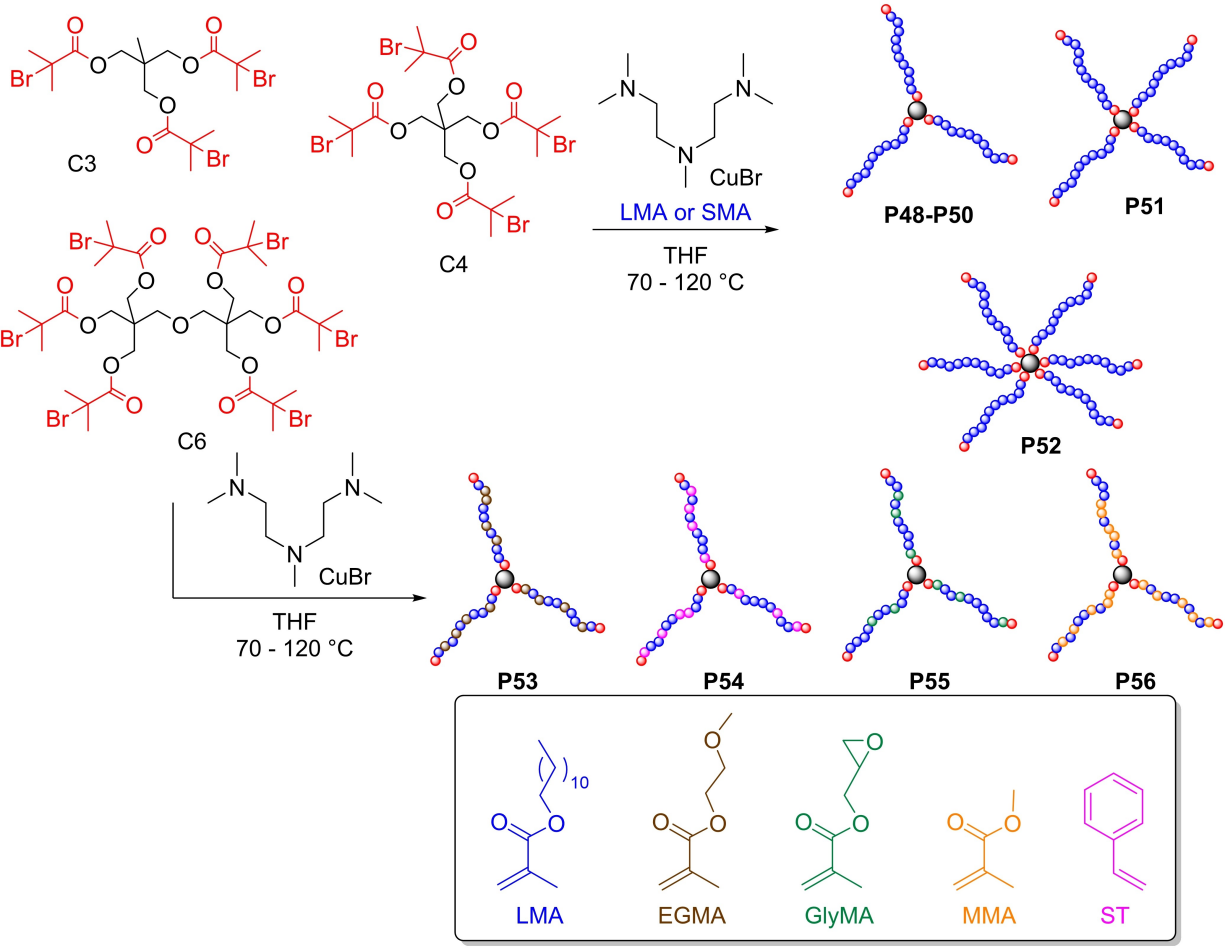
“Core‐first” approach for the synthesis of polymers P48–P56.^[94]^

“Core‐first” synthetic strategies were also reported in literature, as an alternative to the “arm‐first“ approach. Such synthetic approach consists in the synthesis of PAMA‐based arms directly on the core of the final star‐shape architecture. Using the core‐first methodology of Figure 13,

Cosimbescu *et al*. reported the synthesis of homopolymers of LMA or SMA with a well‐defined number of arms imposed by the core molecules used as initiators.[^94^, ^95^] the authors synthesized polymers **P48–P52** using ATRP methodology, which result the best choice for the growing of arms onto a core initiator. ATRP can also have the advantage of avoiding expensive functionalization of starting materials using commercially available RAFT agents or nitroxide derivatives to obtain the core initiators, as required in RAFT and NMP methodologies.[^96^, ^97^, ^98^, ^99^, ^100^, ^101^, ^102^] The authors synthesized two C_3_‐PLMA_n_ homopolymers **P48** and **P49** with different *M_w_* values to study the role of arm length, a C_3_‐PSMA_n_ homopolymer **P50** as comparison platform to investigate the influence of the pendant length on the intramolecular chain entanglement, and other two star‐polymers **P51** and **P52**, having four (C_4_‐PLMA_200_) and six (C_6_‐PLMA_200_) arms respectively, to investigate the role of the arms number on the VMs performance.

The properties of the polymers synthesized are summarized in Table 4. As expected, the trend of polymer **P48–P49** follows the *M_w_* values. In fact, a higher *M_w_* for each of the arms leads to an increase in VI as consequence of a higher values of both KV40 and KV100. Interesting is the comparison of **P49** with **P51** and **P52**, which have more arms. Increasing the number of arms and, at same time, decreasing the *M_w_* of the arms to keep confrontable the *M_w_* of the star‐polymers, brings VI values for **P51** and **P52** very close to the value of **P49**, as well as an enhanced stability to mechanical shear. Star polymers with 3‐arms demonstrated improvements toward reducing friction.

Notably, these improvements in friction may have been inspired by star–star coupling (large molecular weight fractions) or a relatively large polar core. In a follow‐up study, the same group, investigate the role of polarity on the friction reduction achieving dual functional 3‐arms polymer.^[95]^ Authors synthesized 3‐arms copolymers **P53–P56** (Figure 13) using LMA and four polar methacrylates (EGMA, ST, GlyMA and MMA) to investigate the effects of chemical composition and topology towards viscosity and friction reduction (Table 4). In general, the insertion of polar group suppressed viscosity performance (comparison **P49** with **P53–P56**) but enhanced reductions in friction with as much as 40–45 % reduction in coefficient of friction has been demonstrated as compared to the additive‐free oil. The polar monomers utilized have been expected to have interactions with metal surfaces, typical for organic friction modifiers.

Finally, the group of Schmuck and Nyemeyer reported the first application of supramolecular polymers as VMs, and in particular as VII. The concept developed lies at the very foundation of noncovalent interactions in supramolecular chemistry, which are reversible, and temperature dependent. The authors reported novel supramolecular polymers with a reversed viscosity/temperature profile, obtained from a series of ditopic monomers featuring two self‐complementary binding sites,[^103^, ^104^, ^105^] either guanidinio‐carbonyl‐pyrrole carboxylic acids or aminopyridine‐carbonyl‐pyrrole carboxylic acids. At high temperatures, depending on the concentration of the solution, a ring–chain conversion results in the formation of a supramolecular polymer. The reversible transformation can modulate the loss of viscosity at high temperatures, opening applications for innovative systems as viscosity index improvers in working fluids. Indeed, one of the monomers was successfully tested as a promising VII in motor oil.^[106]^

## 3 Conclusion and Outlook

In conclusion, we have introduced the reader to the introductory key concepts and an up‐to‐date summary of research related to the most important class of additives for modern lubrication, the so called “viscosity modifiers” (VMs). Although there are many available polymers used commercially as VMs, research in this field is exponentially growing, especially considering the tremendous advances in polymer synthesis, which nowadays allow the precision construction of very complex macromolecular architectures. In contrast to OCPs and HSDs, PAMA‐based VMs have seen renewed development due to advances in polymerization techniques such as RAFT and ATRP. Indeed, thanks to these technologies, it is possible to develop materials that can perform multiple functions within a formulation.

The field is interdisciplinary, and it needs to bring together specific expertise in polymer synthesis and polymer physics, rheology, and formulation engineering. We have shown how recent studies, in which the synthesis of specific copolymer topologies has been successfully targeted, have demonstrated that some old simplistic models to describe the mechanism of viscosity modulation over temperature, such as Selby's model, are not always fully appropriate. Often such modulation needs to consider the specific chemical characteristics of the species into play and interact with each other, including the most abundant component, the base oil. These are the cases of copolymers obtained via PISA‐RAFT techniques, which gave associative polymers that work as viscosity moderators with supramolecular mechanisms.

Finally, we believe there is ample further space for innovation, especially considering unavoidable considerations about the use of sustainable polymers, for which rheology characteristics can be substantially different. All VMs are essentially dynamic polymers, being able to change, reversibly, their morphology in relation to temperature. The exploitation of dynamic covalent polymer chemistry, in this context, can be certainly forecast as something taken strongly into consideration in the research field.

## Conflict of Interests

The authors declare no conflict of interest.

## Biographical Information

Raffaele Carfora received his Master of Science in Chemical Technology (cum laude) from the University of Milan‐Bicocca in 2018. His master's thesis was focused on the synthesis of novel hydroxy polialogenate phenazines with antimicrobial activity, under the supervision of Professor Antonio Papagni. Following two periods of employment as a laboratory technician in 2020, he commenced doctoral studies with the research group of Dario Pasini at the University of Pavia. His PhD project was focused on the synthesis of novel bio‐based polymer as Viscosity Modifiers. During his PhD studies, he undertook a secondment period at Polymat (San Sebastián, Spain), where he was a member of the research group led by David Mecerreyes. He defended his doctoral thesis in February 2024.

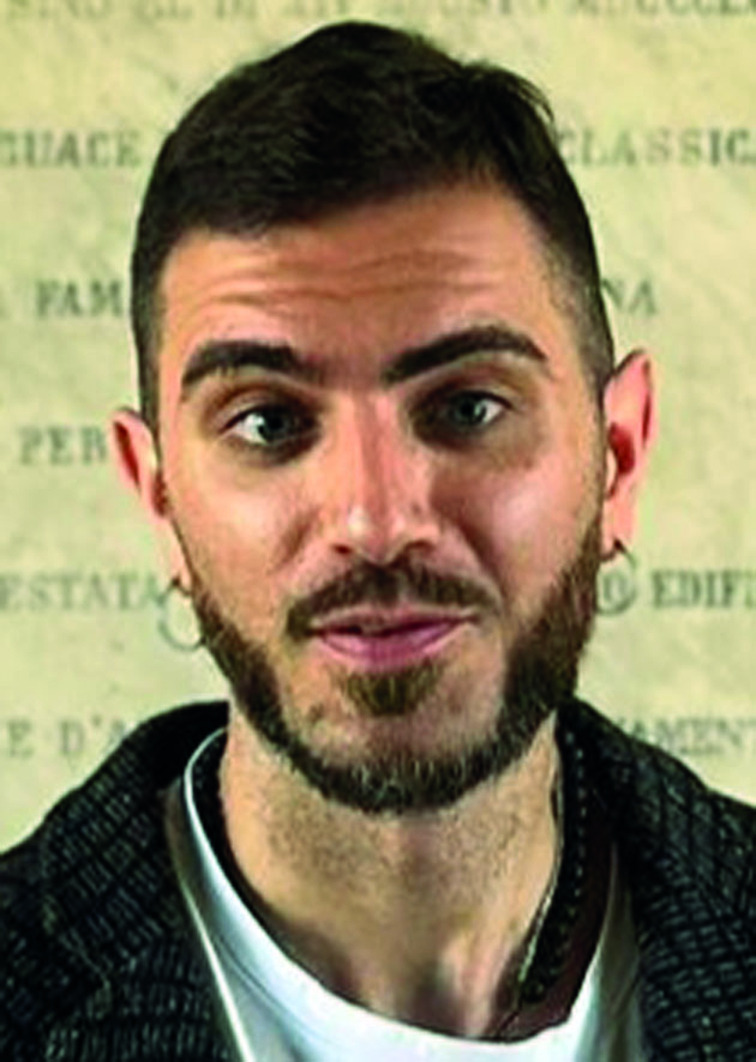

## Biographical Information

Marcello Notari graduated in organic chemistry from the University of Parma, Italy, in 1988. In 1991, he joined Eni, initially working at Enichem in the research and development of chemical intermediates. His focus was on the process development and applications of dimethyl carbonate derivatives, environmentally friendly products. Since 2005, Marcello has been involved in developing additives for lubricants, including polymethacrylates as pour point depressants, various copolymers as viscosity index improvers, dispersants, detergents, friction reducers, and more. His research on additives has closely aligned with the technological evolution of lubricants, requiring environmentally sustainable components that enhance the energy efficiency of machinery and engines. Currently, Marcello is the technical leader and knowledge owner in the additives and lubricants sector.

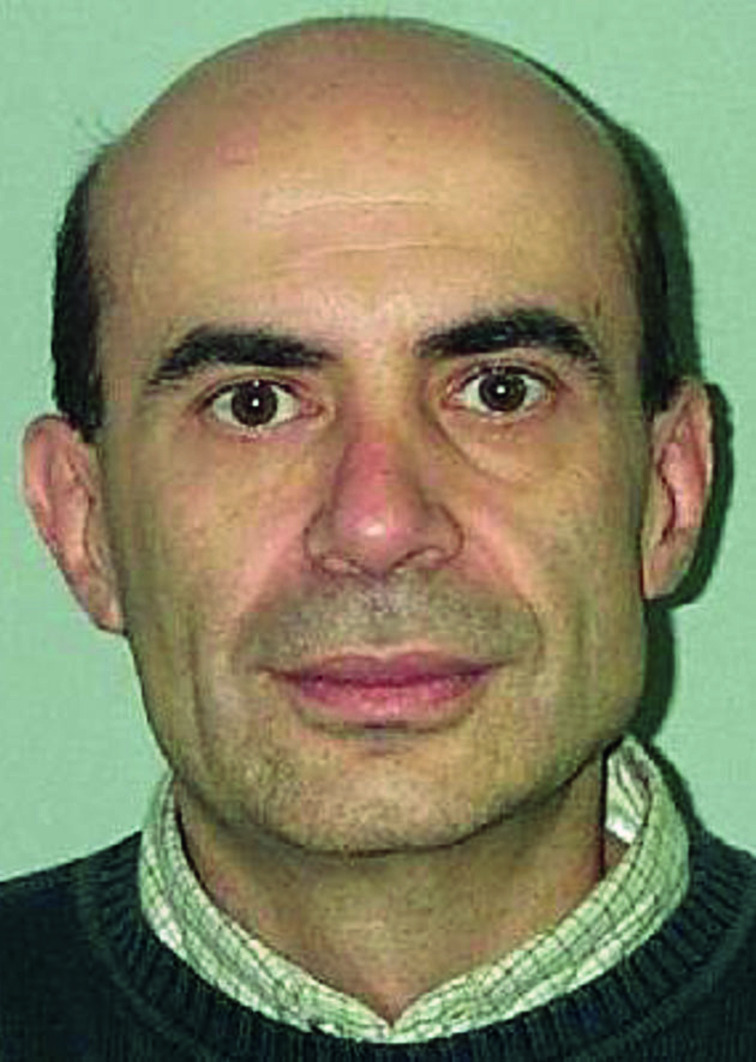

## Biographical Information

Sara Caramia graduated from the University of Bologna in 2017 with a master's degree in industrial chemistry. Since 2018, she has been a researcher at Eni, currently working in the sustainable mobility laboratories. Her role involves the development of advanced additives and lubricants, with a focus on the use of renewable raw materials for the synthesis and formulation of innovative products.

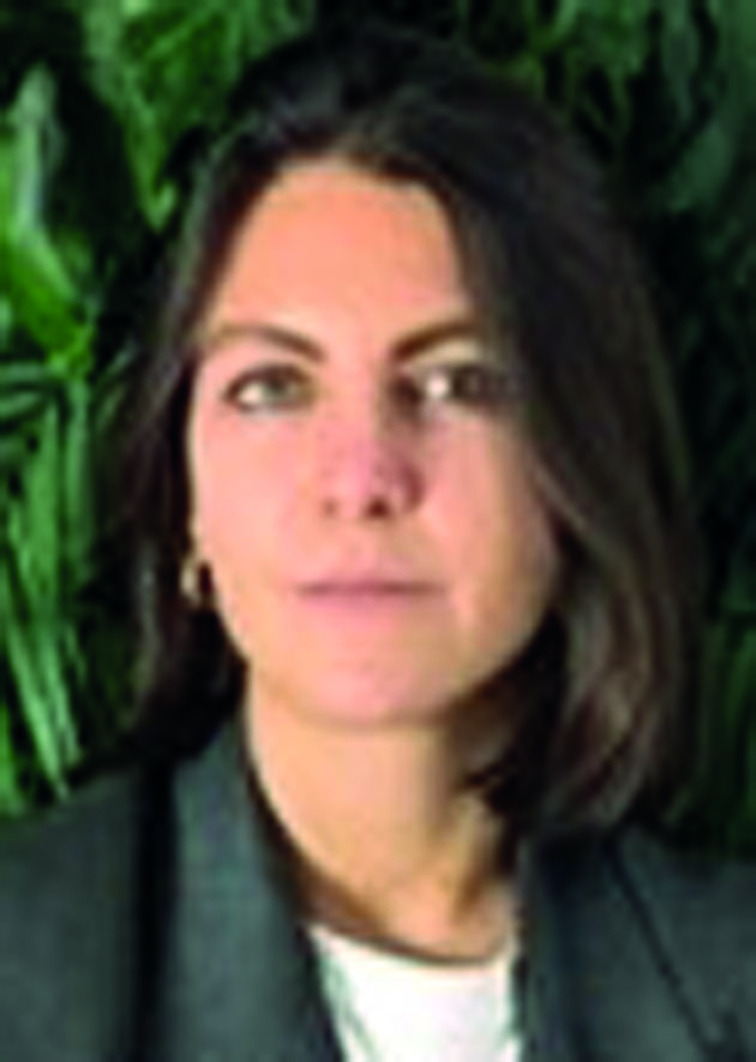

## Biographical Information

After obtaining his organic chemistry degree from the University of Pavia (Italy) and a placement period at Birmingham School of Chemistry (UK), Giulio Assanelli joined Eni in 2011. With a focus on advanced biofuel development initially, his expertise expanded to encompass oil additives synthesis and lubricants formulations. Through his work, he gained valuable insights into the critical relationship between friction reduction and lubricant performance. With a passion for excellence and a deep understanding of lubricant chemistry, Giulio contributes to the development of high‐performance lubricants and innovative additives that enhance energy efficiency, reliability, and sustainability for different commercial applications such as passenger car motor oil (PCMO) and industrial lubricants. Giulio leverages this expertise as an R& D Project Manager, leading innovative projects that drive advancements both in oil additive synthesis and sustainable lubricant technology. Giulio currently leads the R&D unit “Fluids For Mobility”.

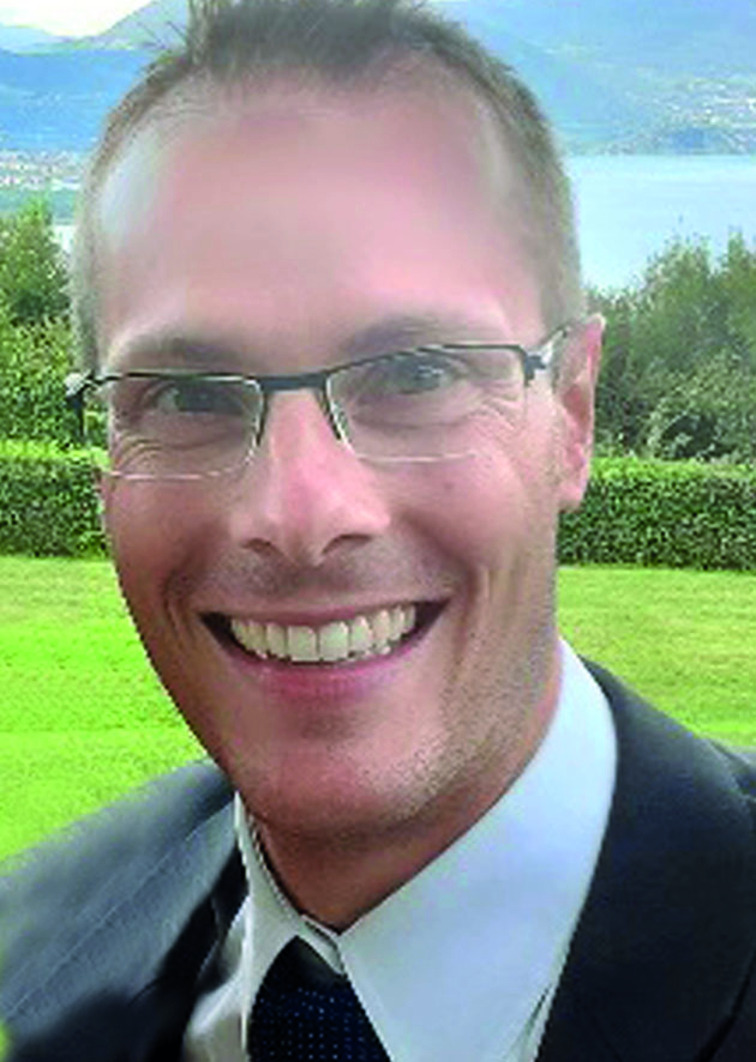

## Biographical Information

Andrea Nitti obtained his MSc Laurea in Chemistry (cum Laude) at the University of Bologna in 2013. He received his PhD in 2017 under supervision of Prof. Dario Pasini at the University of Pavia. During his PhD, he spent a period as a visiting student at MIT in Boston under the supervision of Prof. Timothy M. Swager. Currently he is an assistant professor at the Department of Chemistry of the University of Pavia, focusing on the sustainable organic synthesis of novel oligomeric and polymeric materials for organic electronics, and their processing using advanced manufacturing techniques.

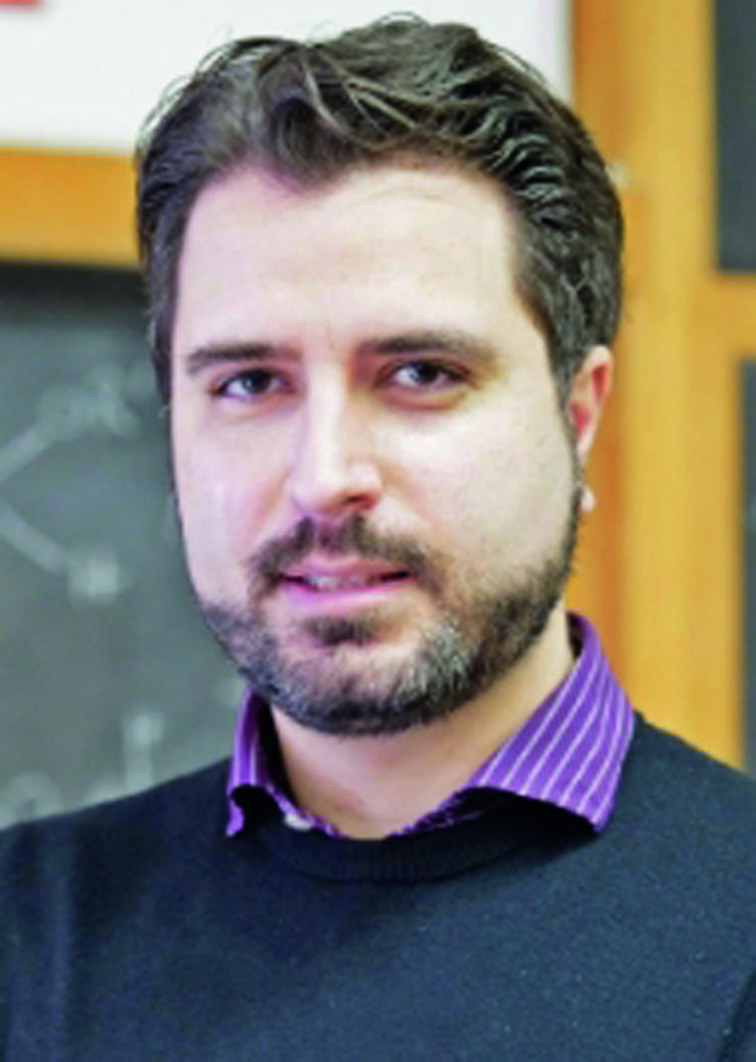

## Biographical Information

Dario Pasini obtained his Ph.D. degree in Chemistry in 1997 from the University of Birmingham, U.K. (advisor: Fraser Stoddart, Nobel Prize in Chemistry 2016). After postdoctoral research at the University of California, Berkeley, U.S.A., in the group of Prof. Jean M. J. Fréchet (1997–1999), he joined the faculty of the Department of Chemistry at the University of Pavia in 2000, where he is now Full Professor of Organic Chemistry. He develops new scientific approaches in the fields of organic, supramolecular, and polymeric materials. He is a Fellow of the Royal Society of Chemistry, and the recipient of a Gutenberg Chair from the University of Strasbourg. He spent periods of research as visiting Professor with S. Matile (Geneva, CH), L. Shimizu (South Carolina, USA) and T. Swager (MIT, USA).